# Neck stabilization through sensory integration of vestibular and visual motion cues

**DOI:** 10.3389/fneur.2023.1266345

**Published:** 2023-11-23

**Authors:** Riender Happee, Varun Kotian, Ksander N. De Winkel

**Affiliations:** Cognitive Robotics, Mechanical Engineering, Delft University of Technology, Delft, Netherlands

**Keywords:** neck, stabilization, sensory integration, sensory conflict, motion sickness

## Abstract

**Background:**

To counteract gravity, trunk motion, and other perturbations, the human head–neck system requires continuous muscular stabilization. In this study, we combine a musculoskeletal neck model with models of sensory integration (SI) to unravel the role of vestibular, visual, and muscle sensory cues in head–neck stabilization and relate SI conflicts and postural instability to motion sickness.

**Method:**

A 3D multisegment neck model with 258 Hill-type muscle elements was extended with postural stabilization using SI of vestibular (semicircular and otolith) and visual (rotation rate, verticality, and yaw) cues using the multisensory observer model (MSOM) and the subjective vertical conflict model (SVC). Dynamic head–neck stabilization was studied using empirical datasets, including 6D trunk perturbations and a 4 m/s^2^ slalom drive inducing motion sickness.

**Results:**

Recorded head translation and rotation are well matched when using all feedback loops with MSOM or SVC or assuming perfect perception. A basic version of the model, including muscle, but omitting vestibular and visual perception, shows that muscular feedback can stabilize the neck in all conditions. However, this model predicts excessive head rotations in conditions with trunk rotation and in the slalom. Adding feedback of head rotational velocity sensed by the semicircular canals effectively reduces head rotations at mid-frequencies. Realistic head rotations at low frequencies are obtained by adding vestibular and visual feedback of head rotation based on the MSOM or SVC model or assuming perfect perception. The MSOM with full vision well captures all conditions, whereas the MSOM excluding vision well captures all conditions without vision. The SVC provides two estimates of verticality, with a vestibular estimate SVC_vest_, which is highly effective in controlling head verticality, and an integrated vestibular/visual estimate SVC_int_ which can complement SVC_vest_ in conditions with vision. As expected, in the sickening drive, SI models imprecisely estimate verticality, resulting in sensory conflict and postural instability.

**Conclusion:**

The results support the validity of SI models in postural stabilization, where both MSOM and SVC provide credible results. The results in the sickening drive show imprecise sensory integration to enlarge head motion. This uniquely links the sensory conflict theory and the postural instability theory in motion sickness causation.

## 1 Introduction

Experimental and simulation studies have shown that vestibular, visual, and proprioceptive information contributes to postural stabilization of the full body in upright standing (1–6), of the unsupported lumbar spine (7, 8); and the neck (9–15). In models of postural stabilization, it is typically assumed that different sensory modalities act as parallel (additive) pathways, with contributions adapted to the task and sensing uncertainty through sensory reweighting (2, 16). However, experiments in humans and primates indicate that multisensory interactions are more complex: Integration of otolith and semicircular vestibular signals shows consistent differences between self-generated or externally imposed motions (17) and involves prior knowledge or experience (18, 19); and interactions between visual and vestibular signals appear to also include assessments of signal causality (20, 21) that can be likened to evaluations of sensory conflict. We are, however, not aware of models of postural stabilization that incorporate more complex aspects of sensory integration.

Postural stabilization has also been linked to motion sickness (MS). According to the sensory conflict theory of MS, conflicting information from different sensory systems or a mismatch between sensation and expectation is what provokes MS (22). This theory has been further refined to state that it is actually a specific conflict between the perceived vertical and expectations thereof that results in MS (subjective vertical mismatch theory) (23, 24). Although conflict-based theories are the most widely accepted explanation of MS, the exact nature of this conflict remains elusive (25). In an alternative theory, the concept of sensory conflict is rejected on the basis that it derives from an *assumption* that sensory signals can be ambiguous or non-specific, which is hypothetical [postural instability theory (26–29)]. These authors argue that patterns of stimulation are unique, when considered across multiple sensory systems, and therefore are not ambiguous. Instead, it is proposed that prolonged periods of postural instability, where humans must exert effort to maintain balance, are the cause of MS. However, it has also been suggested that postural instability is not the cause of MS, but rather that they have a common underlying, perceptual, cause (30, 31). For instance, ref. (32) demonstrated a strong correlation between the individual subjective vertical time constant and motion sickness susceptibility.

Advanced models of vestibular and visual sensory integration have been developed to explain motion sickness causation through sensory conflict (33–36). Such models have also been shown to explain conscious self-motion perception experiments when humans are deprived of visual information. We have shown that the subjective vertical conflict model (SVC) and the multisensory observer model (MSOM) quite well predict motion sickness and motion perception in conditions without vision, but best fits were obtained with different parameter sets tuned either for experimental data from perception studies or for sickness studies (37). We further validated MSOM and SVC models by adding visual perception (38) and showed that the SVC with visual perception of rotation rate (SVC-VR) best predicted sickness, but did not (yet) predict perception in all conditions. Adding visual perception of verticality (SVC-VR + VV) did not improve sickness prediction but somewhat improved perception prediction. The MSOM with visual perception of rotation velocity and verticality (MSOM-VR + VV) best predicted all motion perception experiments used for validation.

In the study presented here, we investigate whether these sensory integration models can also capture postural stabilization in relation to MS. The underlying hypothesis is that unified models of vestibular and visual sensory integration can (at least partially) explain and predict (1) postural stabilization, (2) sensory conflicts leading to motion sickness, and (3) conscious self-motion perception.^1^ We evaluate this hypothesis in an analysis of head–neck stabilization in seated healthy humans, which matches conditions where sensory integration models were validated for motion sickness and self-motion perception. We adopt a biomechanical neck model presented and validated for anterior/posterior stabilization (15) and other directions including frontal impact (39). These articles combined vestibular and visual feedback with joined loops and assumed perfect 3D perception of head orientation in space including verticality and yaw. This assumption of perfect perception is a major simplification, in particular with eyes closed, where verticality perception is confounded by sustained acceleration through the somatogravic illusion (40–43). In the current study, we employ models of sensory integration using physiologically plausible vestibular and visual motion percepts. Vestibular perception consists of otoliths sensing specific force resulting from acceleration and gravity as well as the semicircular canals sensing rotational velocity. Visual perception captures rotation velocities (visual rotation, VR), verticality (visual verticality, VV), and yaw. As motivated in our recent validation study (38), we select the latest versions of MSOM and SVC models of sensory integration of vestibular and visual motion perception and integrate these to capture neck postural stabilization.

Insights and models capturing postural stabilization can be of value in the medical field for research, diagnosis, and treatment and in fields, such as vehicle comfort and impact biomechanics. This study addresses neck postural stabilization in the frequency domain with small loading amplitudes and high bandwidth, illustrating the ability of the models to predict head motion in frequency and amplitude ranges relevant to motion comfort. In addition, we validate the models for a highly dynamic sickening drive eliciting motion sickness.

## 2 Methods

### 2.1 Biomechanical head–neck model

**Figure 1:**
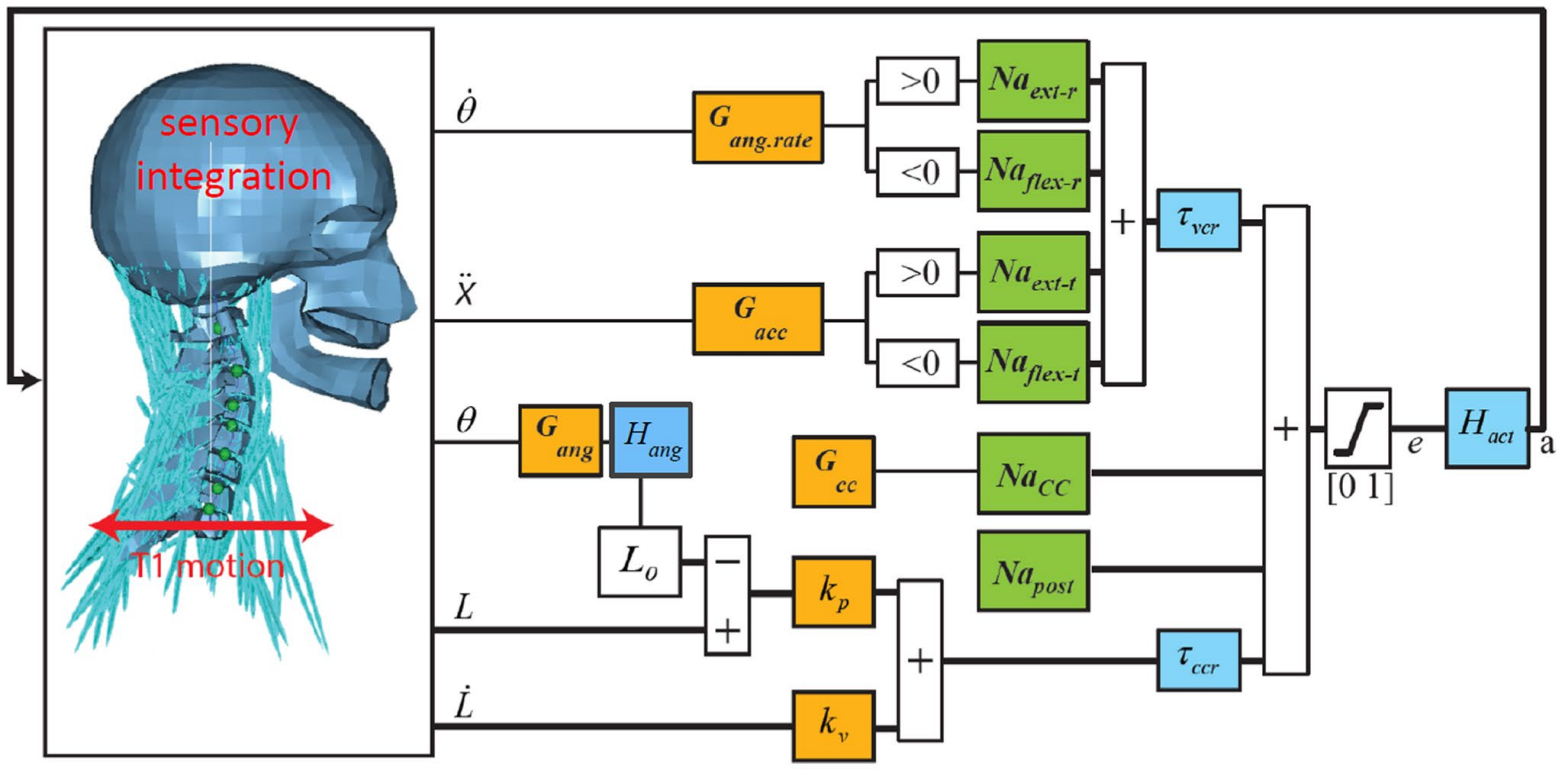
Neural control model of the neck. Trunk motion is applied at the base of the neck (T1). Vestibular and visual motion cues, after sensory integration, result in 3D estimates of head angular rate $\dot{\theta }$ , angle θ , and acceleration $\ddot{X}$ in space. Muscle spindles signal contractile element (CE) length L and velocity $\dot{L}$ . Orange blocks contain the feedback sensitivity (gain) and co-contraction parameters controlling the head angular rate $\dot{\theta }$ , angle θ , and acceleration $\ddot{X}$ with feedback sensitivity parameters *G_ang.rate_, G_ang_*, *G_acc_,* and controlling muscle length with sensitivity parameters *k_p_* (position) and *k_v_* (velocity) *w*here the reference length *L_0_* represents the desired posture, which is modulated to obtain the desired head angles θ . Green blocks are muscle synergy vectors converting scalar control signals to an appropriate activation of multiple muscle segments for flexion (*Na_flex-r_* for rotation and *Na_flex-t_* for translation), extension (*Na_ext-r_* for rotation and *Na_ext-t_* for translation), co-contraction (*Na_cc_*), and postural activity counteracting gravity (*Na_post_*). Blue blocks contain sensory delays for vestibular/visual (*τ_vcr_*) and muscle feedback (*τ_ccr_*) and muscular activation dynamics (*H_act_*) transforming neural excitation (*e*) into muscle active state (*a*). In *head angle control* a first-order low-pass filter *H_ang_* with time constant *τ_ang_* lumps additional delays for visual contributions, neural processing, and control strategies emphasizing lower frequencies. Thick lines indicate multiple signals for all 258 muscle segments. This figure shows anterior–posterior stabilization through neck flexion and extension muscle synergies. Equivalent loops have been added for lateral and yaw motion control.

A wide range of neuromuscular neck models has been presented in the literature, ranging from 1-pivot models (44–46) to detailed multisegment models (47–55) and partial finite element models (56–65). These models were primarily designed for high-severity road accident loading and/or captured only few motion directions. To address these limitations, we adopted a three-dimensional (3D) multisegment non-linear neck model (66–68) extended with a postural controller stabilizing the head–neck system in the presence of gravity and trunk motion [(15, 39); Figure 1].

The neck model contains nine rigid bodies representing the head, seven cervical vertebrae (C1–C7), and the first thoracic vertebra (T1). The eight intervertebral joints allow 3D rotational and translational motion, resulting in a total of 48 degrees of freedom (DOF). Passive joint properties are captured with non-linear force models representing ligaments, intervertebral disks, and facet joints. Muscles (34 muscles, totaling 129 elements per body side) are implemented as line elements based on dissection (69) with ‘via *points*’ connecting muscles to adjacent vertebrae to ensure the muscles take on a curved path during head–neck displacement, and with non-linear Hill type contractile elastic and series elastic dynamics. Gravity is simulated as a 9.81 m/s^2^ gravitational field acting on the skull and the vertebrae. The neck model was validated in passive bending and twist and in isometric loading where the ligamentous spine stiffness, instantaneous joint centers of rotation, muscle moment arms, isometric strength, and muscle activation patterns were in general agreement with biomechanical data (68).

The postural stabilization model, parameter estimation, and validation in anterior–posterior loading can be found in (15). Validation for other directions, including frontal impact, is presented in ref. (39). Feedback loops were added for head lateral motion and yaw, equivalent to the anterior–posterior loops. Lateral loops provide feedback on head roll angular velocity and roll angle in space. Yaw loops provide feedback on head yaw angular velocity and yaw angle in space. As described in the Results section, we explore feedback of head rotational velocity, taking into account the dynamics of the semicircular canals, and explore models of sensory integration to provide feedback of head angles in space. Details on the neck model and muscle dynamics can be found in the Appendix.

The biomechanical neck model was implemented in the simulation software MADYMO 2022. Sensor dynamics, neuromuscular control, delays, and muscle dynamics were implemented in MATLAB R2022b. Euler integration (ode1) was applied with a fixed time step set to 10 μs, resulting in a computation time of approximately 100 times real-time on a 2.8-GHz processor. The ISO coordinate system is applied (x = forward, y = left, z = up).

### 2.2 Models of sensory integration

To capture integration of vestibular and visual motion information, we employed the latest versions of the multisensory observer model (MSOM, see Supplementary Figure A3) (36) and the subjective vertical conflict model (SVC, see Supplementary Figure A4) (33, 34) as described in ref. (38). As described in ref. (38), we use the so-called SVC_I_ model which contains an integrator (dotted box in Supplementary Figure A4d) to process the acceleration conflict Δa. Both models have two vestibular inputs, being the specific force resulting from gravity and acceleration sensed by the otolith organs, and the rotation rate sensed by the semicircular canals. Both models have two visual inputs which are the 3D rotation rate resulting from optical flow, and head orientation consisting of verticality perceived through horizontal structures, such as the horizon or vertical structures such as buildings, and the yaw angle in space. Both models take into account the semicircular dynamics as a high pass filter, which is first order in MSOM, and second order in SVC, while otolith and visual dynamics and delays are ignored. The neck stabilization model also includes “*direct*” feedback of semicircular motion perception (see Section 3.2) using more advanced semicircular dynamics described in the Appendix. For MSOM and SVC, we apply their original vestibular dynamics which were previously used to select the model parameters and to validate these models.

Both MSOM and SVC employ a state estimation approach using an internal model of 6D head motion and semicircular dynamics. Estimated head motion states are adapted using correction loops comparing predicted and actual vestibular and visual motion perception. In this study, we use MSOM and SVC models with different levels of complexity labeled as: NV (no vision or eyes closed) with all visual loops disabled, VR (visual rotation rate) adding visual perception of rotation velocity, and VR + VV also adding visual perception of verticality. The VR option is evaluated to assess the importance of VV comparing VR to VR + VV. Furthermore, the SVC is proposed with only VR by ref. (34) and we recently showed SVC-VR to best predict motion sickness (38).

We applied the MSOM parameters presented in the original publication by Newman (36) and SVC parameters from ref. (33, 34) as summarized in Table 1. We also applied two-parameter sets which we retuned (37) to optimally match motion perception without vision (MSOM-NV-PERC) and motion sickness without vision (MSOM-NV-MS). These two sets include vestibular gains only as they were adapted to conditions without vision.

**Table 1:** Parameters of the MSOM and SVC sensory integration models.

MSOM	Explanation	NV no vision	VR + VV full vision	VR visual rotation rate	NV-PERC perception tuned	NV-MS sickness tuned
*τ_scc_ [s]*	First-order time constant SCC	5.7	5.7	5.7	5.7	5.7
*K_a_*	Gain acceleration vestibular	−4	−4	−4	−3.2	−7.2
*K_f_*	Gain specific force	4	4	4	15.4	0.004
*K_fω_*	Gain specific force to *ω*	8	8	8		8.4
*K_ω_*	Gain *ω* vestibular	8	8	8	2.28	11.2
*K_1_*	Derived parameter	*K_ω_*/(*K_ω_* + 1)	*K_ω_*/(*K_ω_* + 1)	*K_ω_*/(*K_ω_* + 1)	*K_ω_*/(*K_ω_* + 1)	*K_ω_*/(*K_ω_* + 1)
*K_gv_*	Gain verticality visual (VV)		10			
*K_ωv_*	Gain ω visual (VR)		10	10		

SVC		NV	VR + VV	VR	VR + VV high *K_gvis_*	
*τ_scc_ [s]*	first-order time constant SCC	7	7	7	7	
*τ [s]*	first-order time constant LP	5	5	5	5	
*K_ωc_*	Gain ω vestibular	10	10	10	10	
*K_ac_*	Gain acceleration vestibular	1	1	1	1	
*K_vc_*	Gain verticality vestibular	5	5	5	5	
*K_gvis_*	Gain verticality visual (VV)		5		30.2	
*K_ωvis_*	Gain *ω* visual (VR)		10	10	10	

**Figure 2:**
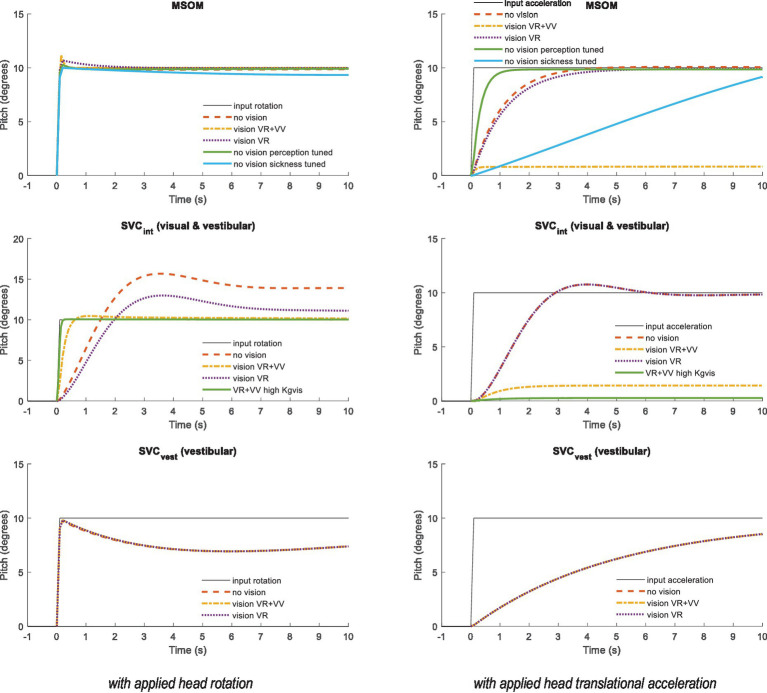
Verticality perception for (from top to bottom) MSOM, SVC_int_ using the integrated verticality v_int_ and SVC_vest_ using vestibular verticality v_vest_. Left: A step pitch rotation with ramp onset (10 degrees in 0.1 s) was applied to the head. Right: A sustained rearward acceleration with ramp onset (0.1 s) was applied to the head with an amplitude of 1.7 m/s*^2^*, which affects the specific force with 10 degrees rotation and therefore induces a steady-state pitch perception of 10 degrees for all models without vision. SVC_vest_ is not affected by vision and therefore the three model lines coincide.

The models can be found in Supplementary Figures A3, A4 in the Appendix. MSOM parameters from ref. (36) are used for NV, VR + VV, and VR. For MSOM additional parameter sets from ref. (37) were applied which were tuned to best capture, respectively, motion perception or motion sickness. SVC parameters for NV, VR + VV, and VR were adopted from ref. (33, 34). For SVC the additional parameter set, “high *K_gvis_*” was tuned to best capture the step response in this study (see text and Figure 2).

The MSOM and SVC do not estimate the head orientation angles in space but estimate the perceived verticality vector *v* in the head coordinate system. We use *v* to derive the perceived head pitch and roll angles in space as:


(1a) $$\mathrm{pitch}=\mathrm{atan}\left(v_{y}/v_{z}\right)$$

(1b) $$\mathrm{roll}=-\mathrm{atan}\left(v_{x}/v_{z}\right)$$

For the MSOM, we use the verticality estimate (Supplementary Figure A3c). The SVC model generates two estimates of verticality, with *v_s_* derived from vestibular otolith and semicircular information, and derived from the internal model integrating vestibular and visual information (Supplementary Figures A4b,d). Therefore, is the more plausible percept, in particular in conditions with vision. However, both percepts may play a role in postural stabilization. Hence, we evaluate both options, which will be further referred to as SVC_int_ using integrating visual and vestibular, and SVC_vest_ using *v_s_* integrating vestibular inputs only. Here, it should be realized that SVC_int_ and SVC_vest_ use different estimates of verticality but are actually derived from the same model.

The MSOM and SVC estimate yaw rotational velocity but do not estimate the head yaw angle. We derive the head yaw angle by 3D integration of the estimated rotational velocity. This integrates vestibular and visual perception of yaw rotation. Such an integration will result in inaccuracy and drift due to sensor imperfections (70, 71). This is realistic for conditions without vision, but availability of vision will correct such imperfections. To describe this, a visual correction loop for the perceived yaw angle in space can be added in future models.

#### 2.2.1 Discrimination of tilt from acceleration

The MSOM and SVC respond to otolith, semicircular, and visual inputs with complex dynamics. Empirical data indicate that the perception of pitch is frequency-dependent. Without vision, slow, low-frequency pitch is perceived as translational acceleration, whereas fast, high-frequency pitch is perceived as such (i.e., rotation), with a crossover frequency of approximately 0.2 Hz (32). Simulations of MSOM and SVC were performed to illustrate these dynamics. Here, we directly prescribed the head motion and did not use the neck model. Figure 2 shows the dynamics of verticality perception by applying a step pitch head rotation (left) and a sustained acceleration designed to elicit the somatogravic illusion (right). Hence, these two cases demonstrate how well the sensory integration models discriminate tilt from horizontal acceleration. The MSOM follows the applied head rotation with a negligible delay and with a precise and stable pitch estimate even without vision. This is expected as the otoliths will sense the constant pitch. For MSOM without vision, the three-parameter sets show marginal differences. The sickness-tuned MSOM-NV-MS deviates a bit but reconverges to 10 degrees after 50 s. Much larger differences between MSOM variants emerge with sustained horizontal acceleration (Figure 2 right). Visual verticality (VV) perception strongly reduces the perceived pitch, while the visual rotation rate (VR) loop hardly affects the result. Without vision, the MSOM predicts persistent pitch perception, matching the somatogravic illusion [e.g., (40–43)]. The sickness-tuned parameters provide a slowly developing somatogravic illusion converging to 10 degrees after approximately 50 s.

##### 2.2.1.1 SVC parameter tuning

The SVC using the integrated vestibular and visual verticality (SVC_int_) follows the applied head rotation with a substantial delay. This delay is smallest with full vision including visual verticality (SVC_int_-VR + VV). As shown in the following sections, this delay hampered effective neck stabilization in conditions without vision. Hence, we explored whether tuning of the SVC parameters could enhance the perception dynamics. This was successful for SVC_int_-VR + VV as illustrated by the lines “high *K_gvis_*” in Figure 2. The increased gain *K_gvis_* contributed to a faster and more precise verticality perception both with head rotation and with sustained acceleration. Tuning other gains marginally affected these responses, and hence, other parameters were left unchanged. For SVC_int_-VR and for SVC_int_-NV, tuning was hardly effective, and hence, results are not shown. SVC_int_ also elicited a somatogravic illusion, which was strongly reduced with vision (Figure 2 right).

The SVC using the vestibular estimate of verticality (SVC_vest_) rapidly follows the applied head rotation and is, as expected, not affected by the visual loops. Due to its fast response, SVC_vest_ is a credible percept, which may effectively contribute to postural stabilization. SVC_vest_ also creates a somatogravic illusion with horizontal acceleration.

The somatogravic illusion (Figure 2 right) develops most rapidly with MSOM followed by SVC_int_ and SVC_vest_. Recent estimates of the time constant of this illusion range between 2 s (42) and 9.2 s (SD 7.17 s) (32), which appear to be matched reasonably well by model predictions. The perception of pitch should be suppressed when vision is available, which is partially achieved by the MSOM and SVC_int_.

**Figure 3:**
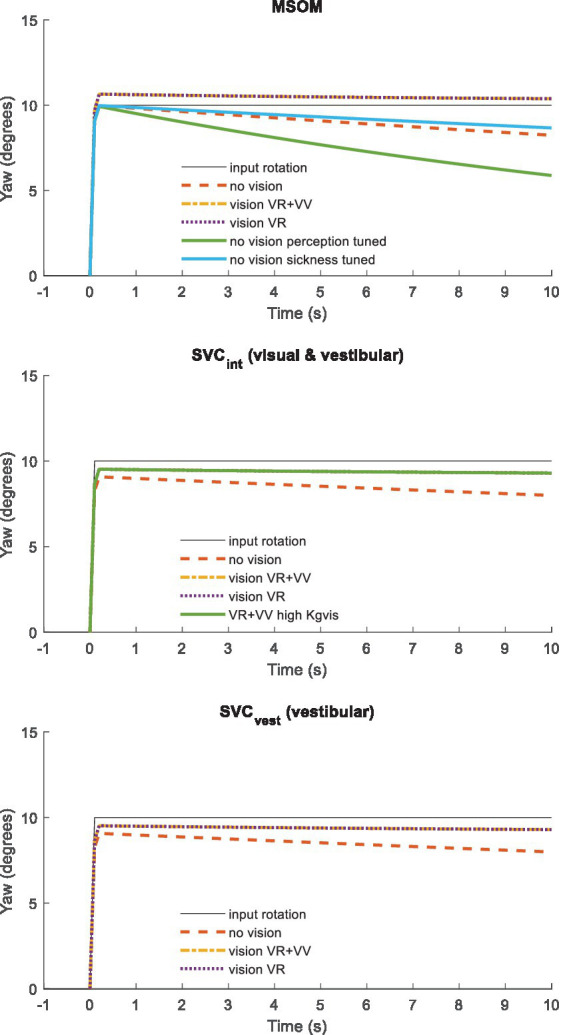
Yaw perception for (from top to bottom) MSOM, SVC_int_ using the integrated verticality v_int_, and SVC_vest_ using vestibular verticality v_vest_. A step yaw rotation with ramp onset (10 degrees in 0.1 s) was applied to the head.

Roll and other tilt directions yield an identical response (not shown) as MSOM and SVC models and the applied parameters are identical for pitch and roll. Figure 3 presents a similar step response for head yaw. Here, all models show a rapid yaw response, which remains with vision. In yaw, the two vision models (VR + VV and VR) respond identically as, as explained above, MSOM and SVC do not use the visually perceived yaw angle and simply integrate the perceived rotation rate. Without vision, the perceived yaw decays to zero where this decay depends strongly on the model parameters as illustrated by the three lines without vision for MSOM. The predicted fading of perceived rotation without vision matches empirical findings where the sense of rotation gradually fades out, with an average time constant of 17.2 s (SD = 6.8 s), in participants rotated around an Earth–vertical yaw axis (32).

### 2.3 Neck postural stabilization

The neck model was validated using postural stabilization data from eight experimental studies with seated healthy adult human subjects as summarized in Table 2. In the first five experiments, subjects were restrained by a harness belt on a rigid seat mounted on a motion platform. In the lateral (Lat) tests, the subjects were also laterally supported with adaptable cushioned plates (73). In all experiments, head motion was recorded in the direction in which seat motion was applied. 3D head motion in both translation and rotation was available for the experiments AP, Lat, Roll, XYZ-compliant, and the slalom. In the anterior–posterior (AP) tests, the T1 translation (base of the neck) was recorded and applied as input to the neck model and used to derive transfer functions from trunk motion to head motion. For Lat, Pitch, and Roll conditions, trunk motion was reported to be close to the seat motion and the seat motion was applied to T1 in the neck model. Checking the transmission from seat motion to T1 in our own data (72, 73), we found gains close to one for trunk horizontal translation and roll, but we also found some phase shifts which shall be considered when interpreting the results. For the yaw conditions, we used recent data (75), which repeated experiments by Keshner (78). The recent dataset was selected as it includes more subjects (17 instead of 7) and describes head global motion as a function of trunk motion recorded at T2. We compared the experimental T2-to-head transmission to the model-based T1-to-head transmission as the model does not include the joint between T1 and T2. The dataset XYZ-compliant was collected on a motion platform with a car seat with a compliant configurable backrest, using the condition with erect posture, high backrest, and eyes open (76). Here, the recorded trunk motion was used to prescribe the T1 motion of the neck model. The slalom was measured in a vehicle on the compliant back seat, and the recorded trunk motion was used to prescribe the T1 motion of the neck model. All reported signals represent motion at the head center of gravity.

**Table 2:** Validation sets for postural stabilization.

Short name	Seat motion and figure showing validation	Bandwidth [Hz]	Vision and instruction sets	Reference
APEO APEC	Anterior–posterior (AP) translation Figure 4	0.2–8	EO = Eyes open, instructed to focus at a marker in front EC = Blindfolded, instructed to maintain a comfortable upright seating position. In both conditions, subjects listened to a science-based radio program to distract them from the stabilization process and minimize voluntary responses.	(72)
LatEC	Lateral (Lat) translation Figure 5	0.15–4	EC = Blindfolded, instructed to maintain a comfortable upright seating position. Subjects listened to a science-based radio program to distract them from the stabilization process and minimize voluntary responses.	(73)
RollEC	Roll (lateral rotation) Figure 7	0.15–4		
PitchVS PitchNV PitchMA	Pitch (anterior/posterior rotation) Figure 8	0.35–3.05	VS=Voluntary Stabilization “required that the subject keep the head-referenced light signal coincident with a stationary target spot” (using a head mounted light spot) NV=No Vision “in the dark subject was given the task of stabilizing the head by imagining the stationary target spot and the head-referenced light signal” MA = Mental Arithmetic “a mental calculation task was provided so that the subject’s attention was removed from the task of stabilization while rotation in the dark was ongoing”	(74)
YawVS YawNV YawMA	Yaw (left/right rotation) Figure 9	0.185–4.11		(75)
XYZ compliant	X, Y, Z loading (sequential) on a compliant seat Figure 6	0.1–12	Erect, Eyes Open, looking forward	(76)
Slalom	Lateral 4 m/s^2^, longitudinal and yaw Figure 10	~0.2	Exterior vision	(77)

## 3 Results

Models of increasing complexity in terms of postural stabilization feedback and sensory integration were fitted to the experimental data. Comparison of model fits can illustrate the relevance of feedback loops and sensory integration across motion conditions. For each condition and model version, the postural control parameters were estimated by fitting the model to the experimental data. The postural feedback gains and co-contraction (see Figure 1) were fitted to optimally match the model response with the human response data in the frequency domain (see Appendix). This generally resulted in a good fit, and hence, the parameters of sensory integration were not fitted and remained as defined in Table 1. As described below, for vertical loading, the data were not very informative and we applied parameter sets estimated for horizontal seat translation from the same dataset, and do not report the model error.

**Figure 4:**
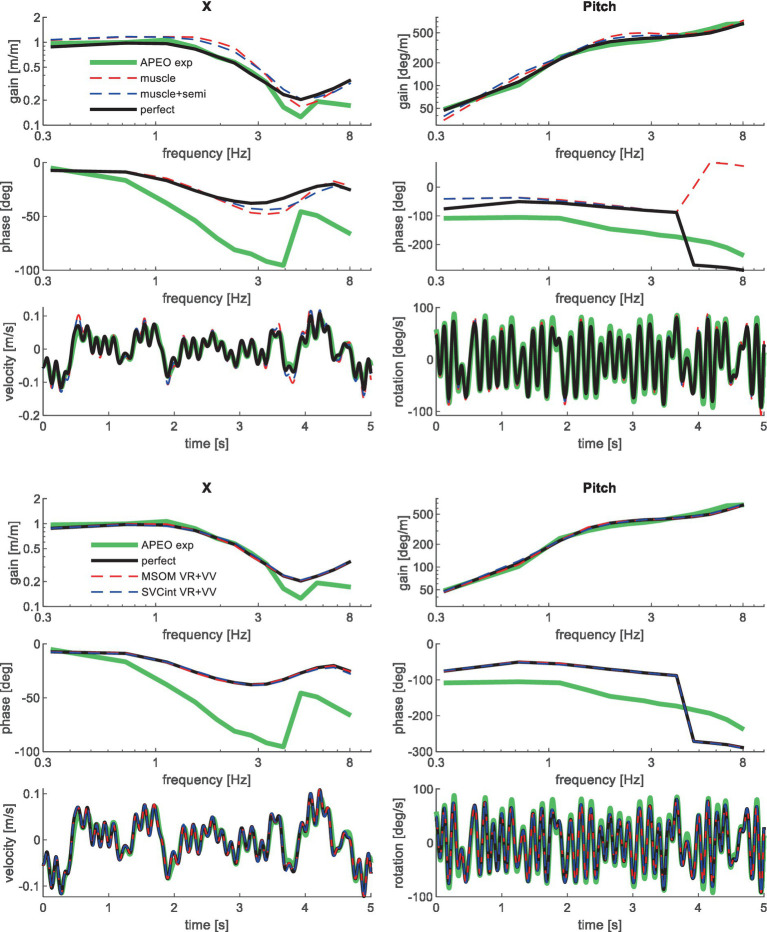
Validation for anterior–posterior seat translation with eyes open (APEO) for muscle/semi/perfect perception (upper 3 graphs), and MSOM and SVC (lower 3 graphs). Only the most relevant models are shown as lines largely coincide.

**Figure 5:**
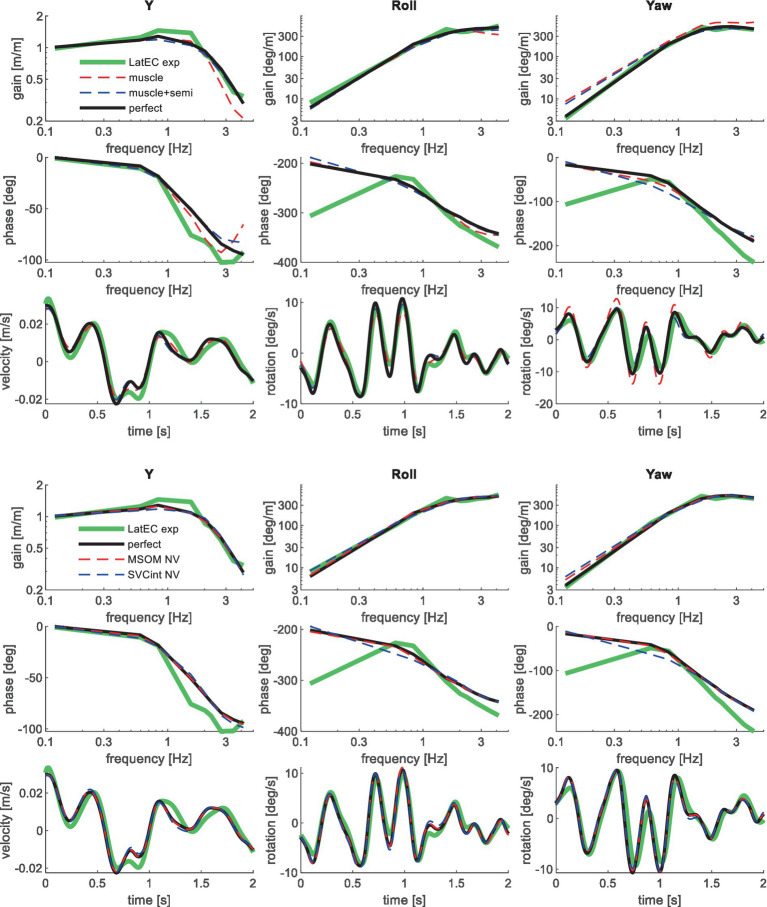
Validation for lateral seat translation with eyes closed (LatEC) for muscle/semi/perfect perception (upper 3 graphs) and MSOM and SVC (lower 3 graphs). Only the most relevant models are shown as lines largely coincide.

**Figure 6:**
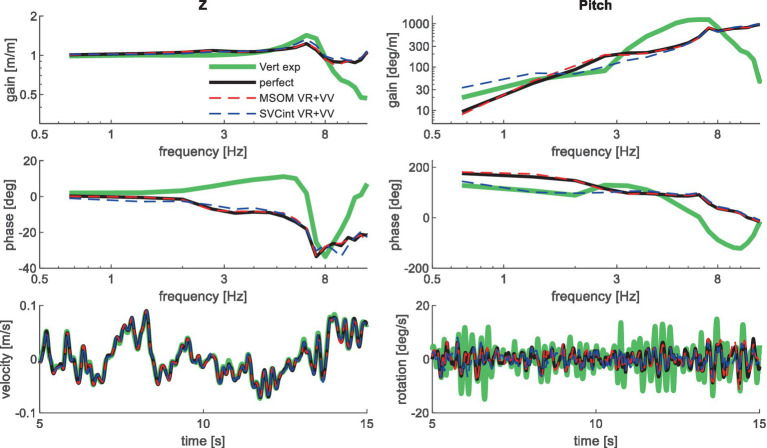
Validation for vertical seat translation on a compliant seat. High-frequency experimental results deviate due to the imprecision of the applied T1 motion, which was based on measured trunk motion. Only the most relevant models are shown as lines largely coincide.

**Figure 7:**
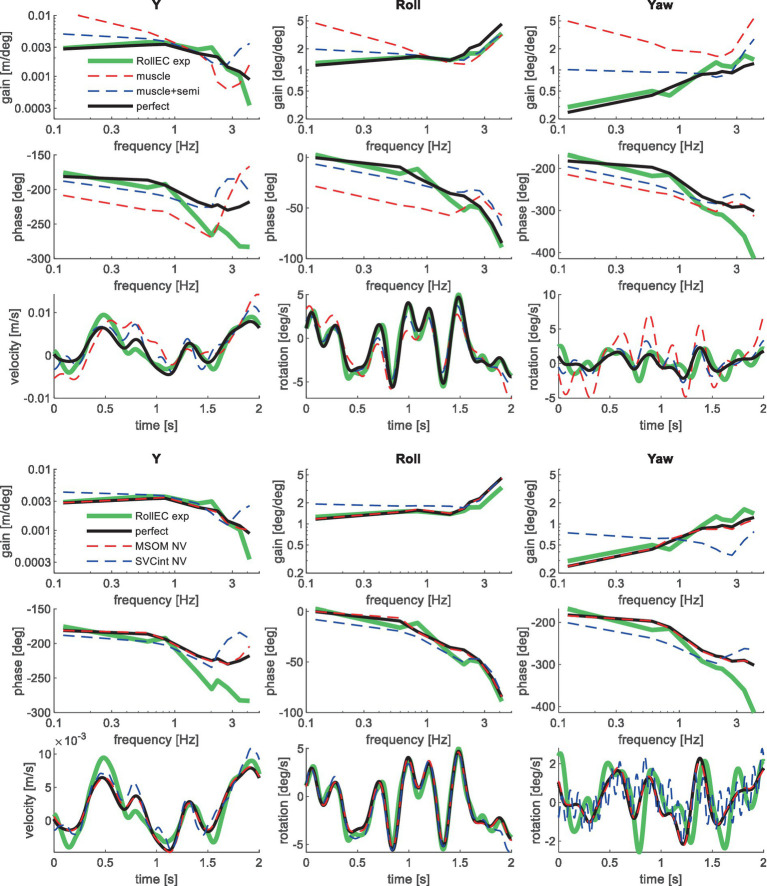
Validation for lateral seat rotation with eyes closed (RollEC) for muscle/semi/perfect perception (upper 3 graphs) and MSOM and SVC (lower 3 graphs). Only the most relevant models are shown as lines largely coincide.

**Figure 8:**
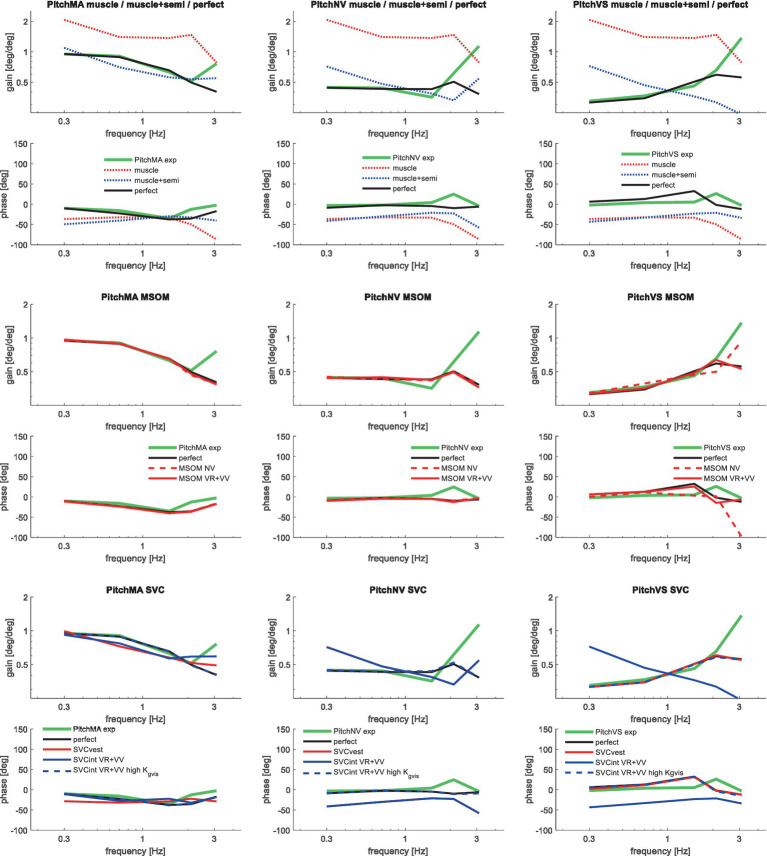
Validation for anterior–posterior seat rotation (pitch) for muscle/semi/perfect perception (upper 2 graphs), MSOM (middle 2 graphs), and SVC (lower 2 graphs) with 3 tasks: PitchMA no vision with mental arithmetic (left), PitchNV no vision active control (mid), PitchVS with vision active control (right).

**Figure 9:**
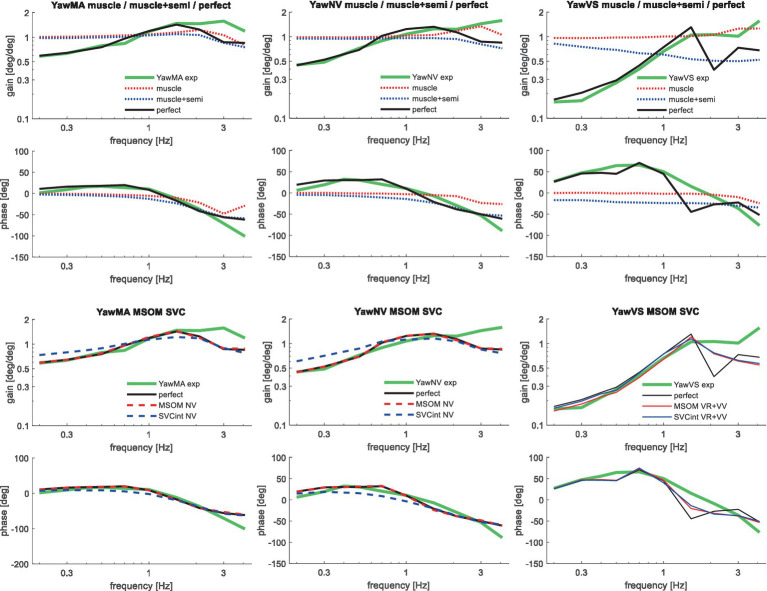
Validation for left/right axial seat rotation (yaw) for muscle/semi/perfect perception (upper 2 graphs), the most relevant MSOM and SVC results as other results overlap (lower 2 graphs) with 3 tasks: YawMA no vision with mental arithmetic (left), YawNV no vision active control (mid), YawVS with vision active control (right).

**Figure 10:**
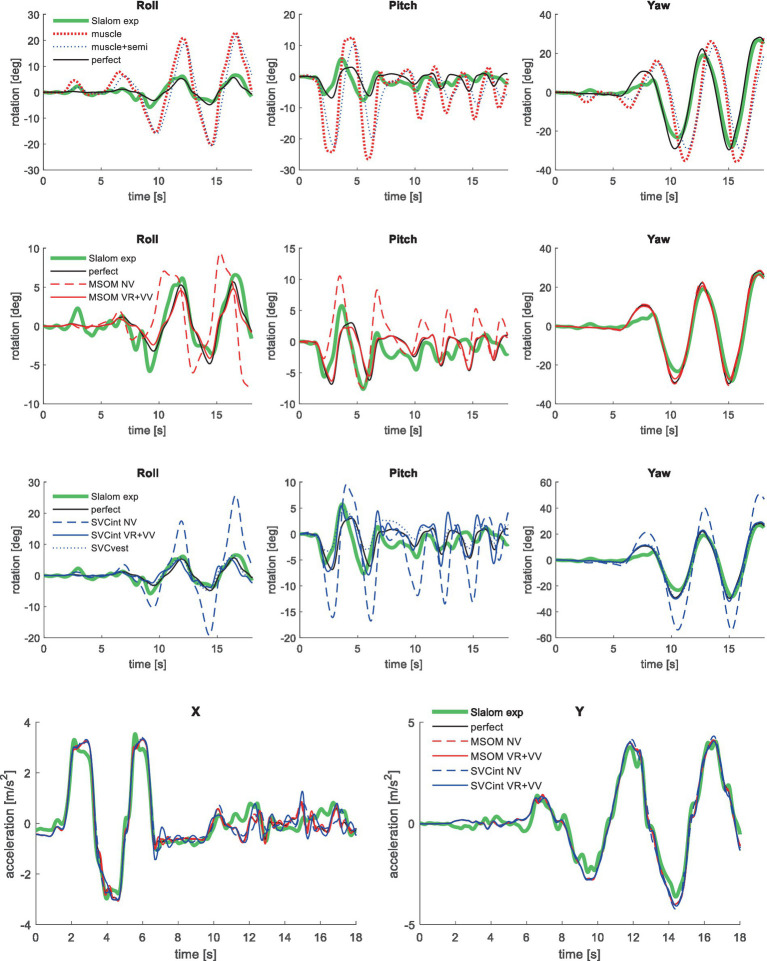
Validation in slalom, showing only the most relevant models, for head rotation (upper 3 graphs) and acceleration (lower graph—responses for muscle and muscle+semi are not shown as they overlap with perfect).

Table 3 shows the resulting model error for postural stabilization and perception models of increasing complexity. Here, the model error was scaled toward the model error assuming perfect perception of verticality and yaw, which shows a (near) optimal fit. Hence, Table 3 allows a rapid comparison of the ability of all models studied to match the human response data. Figures 4–10 show validation results for the most relevant models. Figures 4–9 show the results for the 6-seat motion directions in the frequency and time domain. In each figure, the head response is shown for the perturbed seat motion direction and other relevant head motion directions. For instance, Figure 4 shows relevant head pitch motion in response to AP seat translation and Figure 5 shows head roll and yaw in response to lateral seat translation. Figure 10 shows the validation for the slalom drive in the time domain as for this dataset power is concentrated approximately 0.2 Hz for lateral and yaw motion making the slalom unsuitable for frequency domain analysis.

**Table 3:** Model error in predicting head motion for models of varying complexity in feedback loops (left) and sensory integration (right).

	Feedback loops			Sensory integration-based feedback of head rotation angle (+muscle+semi)											
	Muscle	Muscle +semi	Perfect angle (+muscle+semi)	MSOM NV	MSOM VR + VV	MSOM VR	MSOM NV perception tuned	MSOM NV sickness tuned	SVCint NV	SVC_int_ VR + VV	SVC_int_ VR + VV high *K_gvis_*	SVC_int_ VR	SVC_vest_ NV	OTO angle *τ_ang_* = .03 s	OTO angle *τ_ang_* = 5 s
APEC	1.10	1.00	1.00	#	#	#	#	#	#	#	#	#	#	#	#
APEO	1.94	1.71	1.00	1.10	1.00	1.08	1.21	1.18	1.38	1.10	1.11	1.74	1.03	1.64	*
LATEC	2.47	1.47	1.00	1.04	1.07	1.02	1.05	1.07	1.19	1.16	1.12	1.28	1.00	*	1.31
RollEC	20.63	19.77	1.00	1.13	1.00	1.05	1.21	1.22	3.15	1.55	0.94	1.98	1.01	1.74	3.09
PitchVS	58.37	12.68	1.00	2.80	0.80	2.16	3.96	3.48	*	*	0.97	*	0.98	*	*
PitchNV	26.37	4.13	1.00	0.98	0.99	0.94	1.42	1.08	*	*	1.05	*	1.02	*	*
PitchMA	27.13	4.28	1.00	1.07	1.17	0.99	2.21	1.07	2.90	2.32	0.99	*	0.98	3.31	*
YawVS	14.64	11.23	1.00	0.87	0.94	0.91	2.90	1.00	2.39	0.88	1.03	0.93	0.96	0.96	1.13
YawNV	6.02	5.65	1.00	1.01	1.03	1.03	1.06	1.01	2.28	1.01	1.02	1.51	0.99	1.16	1.14
YawMA	4.26	4.00	1.00	1.00	1.00	1.00	1.06	1.01	1.86	0.97	1.01	1.32	0.99	1.14	0.97
Slalom	2.33	2.19	1.00	1.05	0.96	1.18	*	1.31	1.97	1.09	0.97	1.89	0.93	1.05	*
Average (without APEC)	16.41	6.71	1.00	1.21	1.00	1.14	*	1.34	*	*	1.02	*	0.95	*	*

This table shows errors normalized to the condition “perfect angle perception,” which shows a (near) optimal fit. Colors range from green (good fit), yellow (reasonable fit) to red (poor fit). The feedback loop “muscle” captures muscle length and velocity feedback plus co-contraction (Section 3.1). The feedback loop “semi” also captures feedback of the semicircular canal sensing head rotation velocity (Section 3.2). The feedback loop “perfect angle” also uses the actual head rotation angle in space, assuming perfect perception. The other options use sensory integration models to estimate head rotation.

*not effective: using sensory integration optimal feedback gains of head roll and pitch angle are close to zero.

# not relevant: even with perfect angle perception the optimal feedback gain of head pitch angle is zero.

### 3.1 Muscle feedback

Muscle feedback was implemented representing muscle spindle feedback of relative length and velocity, jointly fitting two parameters for all 258 individual muscle elements. A feedback delay of 13 ms was selected based on ref. (79). Models with muscle length and velocity feedback and without any vestibular and visual motion perception could well stabilize the neck in all conditions. However, a very poor fit of the experimental response was obtained, in particular for conditions with trunk pitch and roll, where the model predicted excessive head rotations (lines *muscle* in Figures 4–10). The model error was on average 16.41 times larger (Table 3) than for the model assuming perfect perception of head verticality and yaw described in Section 3.3. Muscle feedback can apparently well stabilize the head on the trunk, but as expected, muscle feedback cannot effectively reduce head rotation in space in conditions with trunk rotation. Likewise, in the highly dynamic slalom drive, the model with only muscle feedback resulted in excessive head pitch and roll. In some conditions, adding co-contraction (~1%) slightly improved the model fit, but the effects were very similar to the effects of increasing muscle velocity feedback. In the slalom, co-contraction (4%) improved the model fit and allowed higher muscle length and velocity feedback gains while not inducing oscillations. This can be explained by the dynamics of the applied Hill-type muscle model where co-contraction increases muscle damping through the force–velocity relationship and reduces the phase lag of muscular dynamics through preload of the series elastic element.

### 3.2 Muscle and semicircular feedback

Vestibular feedback of head rotational velocity was implemented taking into account the dynamics of the semicircular canals as motivated in ref. (15) and the Appendix using vestibular dynamics from ref. (80). Therefore, we assumed a direct contribution of semicircular perception to head rotation control independent of any model of sensory integration. A vestibular feedback delay of 13 ms was applied based on human studies (81, 82). Adding semicircular feedback was highly effective in reducing head-in-space rotation at the mid-frequencies (lines *muscle + semi* in Figures 4–10), and the average model error was reduced from 16.41 to 6.71 (Table 3). However, at low frequencies and in the slalom, the model still predicted excessive head rotations. We also simulated perfect perception of head rotational velocity, and an estimation of head rotational velocity using the MSOM model integrating semicircular and otolith information. The results for these different estimates of rotational velocity were highly similar and are therefore not shown. The similarity can be explained by the fact that semicircular dynamics show a limited frequency sensitivity in the range from 0.5 to 6 Hz (see Supplementary Figure A2 left in the Appendix) where semicircular feedback most strongly contributed to head stabilization. Even with perfect rotation velocity feedback, the low-frequency head rotation largely exceeded the experimental head rotation in conditions with trunk pitch and roll rotation and in the 0.2 Hz slalom.

### 3.3 Muscle, semicircular, and perfect rotation angle feedback

To enhance the model’s ability to control head orientation at lower frequencies, feedback of head orientation angles was added (verticality and yaw), assuming perfect perception. The vestibular feedback delay of 13 ms was also applied for this loop, combined with a first-order low-pass filter *H_ang_* with time constant *τ_ang_*, lumping additional delays for visual contributions, neural processing, and control strategies emphasizing lower frequencies. For AP, Lat, and Roll, *τ_ang_* was not very sensitive and was set to 100 ms being representative of visual delays. For pitch and the slalom, 30 ms was selected to enhance the model fit, suggesting a strong vestibular contribution to the perception of head rotation angles. For yaw perception *τ_ang_* was increased to enhance the model fit, suggesting a control strategy emphasizing the lower frequencies (YawVS: 400 ms, YawNV: 300 ms, YawMA: 150 ms). The two rotational feedback loops (rate and angle) minimized head rotation in space in all conditions. The only exception is the slalom, where we evaluated both head-in-space and head-in-vehicle control strategies. For the slalom, vehicle roll and pitch were limited, and hence, both strategies yielded a good fit, and we present results for head-in-space control. The slalom showed vehicle yaw up to 30 degrees, and the best results were obtained with head-in-vehicle control for yaw angle while using head-in-space control for the direct semicircular feedback.

Perfect rotation angle feedback led to a very good fit in all validation sets (lines *perfect* in Figures 4–10 and column *perfect angle* in Table 3). Adding feedback of head orientation in space was most effective in conditions with trunk rotation and visual feedback, where the model error was reduced by a factor of 12.68 for PitchVS and 11.23 for YawVS. In the slalom, the model error was reduced by a factor of 2.19 due to reduced head pitch and roll and due to a better alignment in time for head yaw, where head-in-vehicle control (line *perfect*) yielded much better results than muscular head-on-trunk control (line *muscle*).

The assumed perfect perception of head orientation is plausible in conditions with visual feedback. The condition APEC without visual feedback was well described without such feedback, but for all other conditions without visual feedback, rotation angle feedback was needed to match the experimental data. To provide a neurologically plausible control model in conditions with and without vision, we explored models integrating vestibular and visual sensory information as explained in the following sections.

### 3.4 Otolith (OTO) feedback

As a first step, we assumed verticality perception simply using low-pass-filtered otolith information (OTO) to control head pitch and roll. Such a low-pass filter aligns with the concept of otolith information to reflect acceleration at high frequencies and verticality at low frequencies (40–43, 83–86). A first-order low-pass filter was used with time constants *τ_ang_* between 0 and 10 s. With low values (*τ_ang_* ≤ 0.5 s) OTO feedback improved results for the slalom and RollEC, but the most demanding Pitch cases showed marginal improvement (see results for *τ_ang_* = 0.03 s in Table 3). As compared to perfect perception of pitch and roll, with OTO feedback, gains had to be reduced to prevent oscillations and this was even more detrimental with larger *τ_ang_* (see the results for *τ_ang_* = 5 s in Table 3). Apparently, low-pass-filtered OTO feedback is hardly usable to dynamically control head pitch and roll. Results for Yaw conditions were hardly affected by OTO feedback, which is not surprising as the presented OTO results simply assumed perfect yaw perception.

### 3.5 MSOM and SVC

The above results illustrate that a more advanced estimation of head rotation angles is needed. This was achieved using the MSOM and SVC models of sensory integration described in Section 2.2, which provide plausible estimates of verticality (head pitch and roll) and yaw. Instead of perfect perception, now head verticality and yaw derived using MSOM or SVC were used for head orientation feedback. Feedback of head rotational velocity remained based on direct semicircular feedback as described in Section 3.2. The feedback delays and time constants introduced above were kept as MSOM and SVC do not include delays. Future models could redistribute delays and time constants across feedback and sensory integration models.

Table 3 shows that in particular the roll, pitch, and slalom conditions are well captured by several sensory integration model variants but not by others. The AP and Lat cases are hardly sensitive because the gain of angular feedback is limited. In the Yaw cases angular feedback is very important, but apparently, all MSOM and SVC model variants adequately predicted head yaw. The only exception is SVC_int_-NV where the head is not well stabilized leading to complex 3D head motion including substantial roll. As only yaw data were available, we could not assess the validity of head roll and pitch in response to trunk yaw. The similarity of results for MSOM and SVC variants concurs with Figure 3, which also shows MSOM and SVC model variants to yield almost identical responses in yaw. Head yaw was derived from MSOM and SVC through integration which is overly simplistic, and the scope for further validation and improvement of the MSOM and SVC models in predicting head yaw will be addressed in the discussion.

The MSOM model with full vision (MSOM-VR + VV) provided a good fit in all conditions and was generally close to results with perfect perception of verticality and yaw (Table 3). The MSOM no vision (NV) model well captured all conditions without vision. In most conditions, MSOM results were not very sensitive to the applied visual loops. However, the PitchVS case was well fitted by the MSOM-VR + VV model and not by the MSOM-NV and MSOM-VR models, highlighting the importance of visual verticality perception. In the slalom drive, the MSOM with full vision (MSOM-VR + VV) yielded realistic head rotations, whereas the MSOM-NV yielded larger head pitch and roll. We did not find similar experimental driving data comparing external vision to no vision, so we collect such data in ongoing experiments. The MSOM-NV results were sensitive to the applied parameters in roll, pitch, and slalom where the perception-tuned parameters and the motion sickness-tuned parameters (37) were less effective in stabilizing head rotation. This is most apparent in the two trunk tilt conditions without vision (RollEC and PitchNV) where the standard MSOM parameters (MSOM-NV) provide a good fit and the perception-tuned and sickness-tuned MSOM parameters show a substantial model error. The SVC provides two estimates of verticality (see Section 2.2). SVC_int_ using vestibular and visual information shows a considerable time lag (Figure 2), in particular without vision. Hence, SVC_int_-NV was hardly effective in controlling head pitch and roll. For SVC_int_-NV, the head pitch and roll angle feedback gains had to be substantially reduced in order to achieve stability, and in the PitchVS, and PitchNV cases, the SVC_int_-NV model did not improve results. This can be seen comparing columns in Table 3, where the model error for SVC_int_-NV shows no improvement compared with model *muscle + semi,* which has no feedback of head rotation angle. The SVC with full vision (SVC_int_-VR + VV) provided good results in most conditions with the published parameters (33, 34). However, results in pitch improved substantially (Table 3) with the high *K_gvis_*, which was tuned for the step pitch perception simulation in Figure 2. Hence, SVC_int_-VR + VV well captures conditions with vision, but SVC_int_-NV poorly describes conditions without vision. The much faster vestibular estimate of verticality SVC_vest_ provides good results in most conditions, which are very close to results with perfect perception of verticality. Apparently, SVC_vest_ is highly effective in stabilizing head verticality and is suitable for capturing conditions without vision. Possibly SVC_int_ and SVC_vest_ are jointly used to control head verticality. However, the current data are not suitable to validate such separate contributions as a good fit was already obtained with SVC_vest_. SVC_vest_ is not designed to estimate yaw, and we estimated yaw by 3D integration, which proved to be effective in the current validation.

### 3.6 Six degrees of freedom neck dynamics

**Figure 11:**
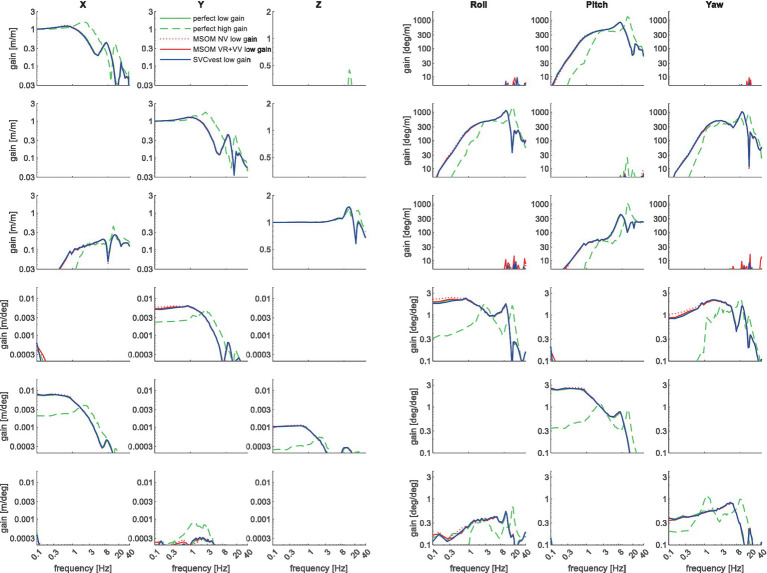
Six degrees of freedom head–neck model response. Rows describe applied T1 motion conditions from top to bottom: AP, lateral and vertical translation roll, pitch, and yaw rotation. Columns describe the corresponding head motion. Lines represent different sensory integration models. The line “perfect high gain” uses the high feedback gains estimated for the slalom with perfect estimation of head rotation. All other lines use low feedback gains jointly estimated for the horizontal loading conditions APEO and Lat. The high gain substantially affected the results, whereas the results for perfect perception, MSOM, and SVC hardly differed.

The above results show model fits for head motion in the applied seat motion direction, and several other (interacting) head degrees of freedom. Available datasets were limited in bandwidth, but the model allows us to extrapolate the human response to a larger frequency range. Figure 11 shows such results for all six-seat perturbation and head response directions. In all cases, motion was applied directly at T1. As the model and the adopted posture are left/right symmetric, several interaction terms in Figure 11 are zero; for instance, AP motion (top row) does not induce lateral, roll, and yaw motion. Other interactions show zero gains as the linearized transmission is zero, but non-linear behavior will induce higher harmonics. For instance, lateral seat motion (second row) will induce some vertical head motion, but this will be identical to left or right seat motion, leading to a zero linearized transmission.

The three upper left diagonal cells in Figure 11 show that T1 translation induces head motion in the corresponding direction with a gain close to one at low frequencies. At mid-frequencies, some amplification (gain>1) is shown for all three translation motion directions, in particular with high feedback gains. As expected the high feedback gains effectively reduce head rotations in all three directions (columns roll, pitch, and yaw) in all loading conditions (all rows). The sensory integration models have limited effects.

### 3.7 Motion sickness prediction

Table 4 shows the sensory conflict between the perceived and estimated verticality vector in the slalom. This conflict is limited for MSOM given the magnitude of applied vehicle acceleration of 4 m/s^2^. Larger conflicts are found for SVC, indicating non-perfect estimation of verticality (as associated with motion sickness causation). For MSOM, the conflict is slightly larger with full vision (VR + VV) as compared to NV, which is unrealistic. The SVC predicts the expected larger conflict with eyes closed (NV) as compared to full vision (VR + VV), which aligns with higher sickness being driven without vision (77, 87). The SCV_int_-VR + VV model with high *K_gvis_* improves the model fit (Table 3) but hardly affects the conflict (Table 4). The SVC_int_-VR is promoted as model to predict sickness in several publications and was indeed found to best predict sickness in our evaluation of MSOM and SVC models (38). Comparing SVC_int_-VR to SVC_int_-NV also shows the expected trend of a larger conflict without vision.

**Table 4:** Sensory conflict and head roll for the slalom.

	MSOM NV	MSOM VR + VV	MSOM VR	SVC_int_ NV	SVC_int_ VR + VV	SVC_int_ VR	SVC_int_ VR + VV high *K_gvis_*
Conflict rms [m/s^2^]	0.52	0.69	0.54	1.367	0.351	0.732	0.348
Head roll rms [deg]	3.71	1.87	4.32	9.18	2.13	9.56	2.13

For the SVC, Table 4 shows more head roll with a larger sensory conflict comparing SVC_int_-VR + VV to SVC_int_-NV and comparing SVC_int_-VR to SVC_int_-NV. This larger head roll results from an inaccurate estimation of the head rotation, making feedback of the estimated head rotation less effective. This shows that inaccurate perception can jointly induce sensory conflict and postural instability, joining both the sensory conflict and the postural instability theory of motion sickness causation.

## 4 Discussion

A biomechanical neck model was uniquely extended with postural stabilization using SI of vestibular (semicircular and otolith), and visual (rotation rate, verticality, and yaw) cues using the multisensory observer model (MSOM) and the subjective vertical conflict model (SVC). The ability of the combined model to capture postural stabilization and motion sickness causation was evaluated using existing empirical datasets, including 6D trunk perturbations and a slalom drive inducing motion sickness.

### 4.1 Insights gained in neck postural stabilization

The neck model with postural stabilization well matches experimental head translation and rotation responses. When omitting vestibular and visual perception, muscular length and velocity feedback could stabilize the neck in all conditions. Muscle feedback can stiffen the neck supporting a head-on-trunk control strategy. This provided a reasonable fit for low-amplitude horizontal seat translation conditions (AP and Lat). This also concurs with observations in vestibular loss patients where “there are no dramatic differences between patients and controls” in conditions similar to APEC (88). However, this resulted in excessive head rotations in conditions with trunk pitch and roll and in the highly dynamic slalom drive. Here, realistic results could only be obtained by adding two rotational loops: *G_ang.rate_* providing direct feedback of head rotation velocity as sensed by the semicircular organs using vestibular sensitivity functions (80) and *G_ang_* representing feedback of rotation angles (verticality and yaw). These loops support a head-in-space control strategy for all conditions studied, with an exception for yaw in the slalom where visual feedback aligned the head with the vehicle driving direction. Direct feedback of 3D head rotational velocity sensed by the semicircular canals effectively reduced head rotations at the mid-frequencies. Realistic head rotations at low frequencies were obtained by adding sensory integration (SI) based feedback of head rotation in space (verticality and yaw). This supports the validity of the MSOM and SVC SI models in postural stabilization. A good fit was also obtained assuming perfect perception of verticality and yaw. Hence, this assumption of perfect perception is adequate when developing models predicting head motion. However, this assumption is not justified when developing models explaining neck postural stabilization, where SI models can help unravel the role of vestibular and visual information.

Conditions with small amplitude trunk translation (AP and Lat) were hardly sensitive to the applied SI model. This can be explained by the low gain for angular feedback estimated for these conditions, making the response insensitive toward the selected pitch and roll perception model. In experimental conditions with trunk pitch and roll and in the highly dynamic slalom, high angular feedback gains were needed to fit the data, and head rotation varied strongly with SI model type and parameters. In particular, the pitch condition with three instruction and vision conditions (VS, NV, and MA) was highly suitable for validation. For roll, only one instruction was tested (NV), and it may be interesting to collect VS and MA data. MSOM and SVC both use the same parameters for pitch and roll and therefore respond identically to pitch, roll, and other tilt directions. However, quasistatic experiments show more precise tilt perception in roll (89). In yaw, three instruction and vision conditions (VS, NV, and MA) were tested, and like in pitch, feedback of head rotation angles in space proved essential to match the experimental data. In yaw, all MSOM and SVC variants except SVC_int_-NV well captured the data. However, MSOM and SVC have not been designed for yaw angle perception and are only validated for yaw rate perception for the cases of earth–vertical and off–vertical axis rotations at a constant yaw rate (38). Here, it was observed that only the models with visual rotation (VR and VR + VV models) are able to simulate human yaw rate perception. For this study, we extended the MSOM and SVC to estimate the yaw angle through the integration of yaw rotation velocity. This well captured the current data but will be inadequate in cases with sensory imperfections, calling for further validation and addition of a visual yaw angle perception loop.

The MSOM-VR + VV with full vision well captured all conditions, whereas the MSOM-NV without vision captured all conditions without vision. This illustrates that the MSOM is a plausible model to capture and explain neck postural stabilization. The SVM provides two estimates of verticality, with a vestibular estimate SVC_vest_, and an integrated vestibular/visual estimate SVC_int_. The vestibular SVC_vest_ well captures all conditions, including those with vision. This makes SVC_vest_ a plausible contributor to head–neck stabilization but fails to explain the role of vision. The integrated SVC_int_ shows plausible results but follows the actual head rotation with a substantial delay, in particular without vision. With vision, this delay could be effectively reduced increasing the verticality perception gain *K_gvis_*. This resulted in a faster and more precise verticality perception both with head rotation and with sustained acceleration (Figure 2) and a better fit of human head motion data (Table 3). With high *K_gvis_*, the SVC approximates perfect verticality perception, which is actually equivalent to a direct feedback of the visually sensed verticality. Without vision, tuning of the SVC parameters was not effective, and the slow response of SVC_int_-NV led to oscillations with high angular feedback gains, and a poor fit, in particular with trunk pitch. Hence, SVC_int_ alone cannot explain head–neck stabilization without vision. However, SVC_int_ may well complement SVC_vest_, and with such a combined approach, the SVC will also be a plausible model to capture and explain neck postural stabilization.

MSOM and SVC were tested with full vision (VR + VV) using both visual rotation rate (VR), and visual verticality (VV) as well as with rotation rate only (VR). Adding the VV loop enhanced the model fit both for MSOM and SVC showing the relevance of visual verticality perception in head–neck stabilization.

Muscle feedback stabilizes the head on the trunk. Muscle feedback also proved essential for the stabilization of the individual intervertebral joints and to prevent neck buckling. Without muscle feedback, static stability could not be achieved, resulting in excessive static flexion or extension of the individual neck joints and the entire neck (15). Neck muscle co-contraction was estimated to be up to 1% of maximal muscle activation and 4% in the slalom. Co-contraction contributed to head-on-trunk stabilization up to 1 Hz and allowed higher feedback gains in the slalom. This highlights a relevant contribution of neck muscle co-contraction, in particular in high-acceleration conditions.

### 4.2 Modulation of postural stabilization

Experimental studies have shown the ability of the central nervous system (CNS) to modulate neck afferent feedback in response to changing external environments (9, 90–94). We demonstrated modulation of neck afferent feedback with the frequency bandwidth of anterior–posterior trunk perturbations (72), with modest effects of the presence of vision. The neck model enabled the estimation of postural control parameters for these conditions (15). Control strategies employed during low-bandwidth perturbations most effectively reduced head rotation and head relative displacement up to 3 Hz, while control strategies employed during high-bandwidth perturbations reduced head global translation between 1 and 4 Hz. This indicates a shift from minimizing head-on-trunk rotation and translation during low-bandwidth perturbations to minimizing head-in-space translation during high-bandwidth perturbations. This modulation of control may well be beneficial in terms of comfort, limiting the transfer of 1–4 Hz horizontal seat motions to the head, where comfort standards for whole body vibration attribute considerable weight to these frequencies (95).

The current study evaluated fundamentally different motion conditions and tasks resulting in a stronger modulation of postural gains. High gains for pitch angle were needed in the pitch and slalom conditions (gains were APEO:0.5; PitchMA:1.9; PitchNV:4.8; PitchVS:7.1; slalom:5.2). Similarly, the gains for roll angle were modulated strongly (LatEC:0.6; RollEC:1.3; slalom:6.5). Figure 11 illustrates the importance of feedback gain modulation across 6D perturbation and response directions, where the “high gain” response matching the dynamic slalom drive differed profoundly from the response with low gains estimated for low-amplitude horizontal acceleration. This postural feedback modulation may be beneficial affecting comfort and muscular effort, and we are currently exploring optimal control strategies to explain and predict modulation of postural stabilization.

The SI models have now been applied with the published parameter sets and with two alternative parameter sets for MSOM (NV-PERC and NV-MS) and one for SVC (VR + VV high *K_gvis_*) (Table 1), showing marked effects of SI parameters on postural stabilization. The SI parameters may also be modulated with motion conditions and tasks. As the current data were already well captured, we did not attempt to fit the SI parameters with the current validation sets. Here, perception experiments are presumably more informative and suitable to validate SI models and parameters (37, 38).

### 4.3 Sensory conflict, postural instability, motion sickness, and motion perception

This study uniquely links models of sensory integration to postural stabilization, with a comprehensive validation for postural stabilization complemented with an exploration of motion sickness. As expected, in the sickening slalom, SI models could not precisely estimate the actual head rotation (verticality) resulting in sensory conflict. Thus, our results support the sensory conflict theory in motion sickness causation. The SVC predicted larger conflicts than the MSOM, which provides a close to perfect prediction of verticality, indicating the SVC to be more promising for sickness prediction. When removing vision, only the SVC predicts the expected increased sickness (77, 87). These results are in line with our recent study (38) showing SVC to be more suitable for motion sickness prediction, whereas MSOM best captured motion perception. We feel that both MSOM and SVC can be enhanced to further explain postural stabilization, motion perception, and motion sickness. Here, we will aim for common sensorimotor integration models but will also take into account evidence toward partially different processes (96).

Our results also show that imprecise sensory integration can enlarge head motion. This shows that inaccurate perception can jointly induce sensory conflict and postural instability, relating both the sensory conflict and the postural instability theory in motion sickness causation.

### 4.4 Limitations and future study

The current models assume group-based perception and postural stabilization parameters to fit group-based postural stabilization data. However, individuals show marked differences in kinematic, perception, and motion sickness responses. Interpersonal variability in model parameters, or even fundamental differences in neural processing, may potentially explain such individual differences in responses, and link the domains of postural stabilization, motion perception, and motion sickness. For instance, individual motion perception time constants were recently shown to relate to sickness (32). Therefore, the next challenge will be to estimate individual parameters for perception, postural stabilization, and motion sickness susceptibility (97). This can combine dedicated experiments measuring perception, postural stabilization, and sickness on a substantial pool of participants, thereby disclosing relations across these domains and explaining individual differences. The validation can also be extended in frequency range, in particular for pitch and yaw where current data are limited to 3 Hz while Figure 11 shows effects of feedback up to approximately 8 Hz. Effects of SI models and parameters are most apparent at low frequencies (<1 Hz) and can be studied using low-frequency or quasistatic experiments.

The current model is deterministic with a non-linear Hill-type muscle model, non-linear passive structures, and linear idealized models of sensors and sensory integration. Stochastic and more detailed non-linear models may further explain sensory integration and neck stabilization including postural sway. Vestibular dynamics are described as lumping regular and irregular afferents (see Appendix), using a linear second-order semicircular function for direct feedback, a first-order function in MSOM, and a second-order function in SVC, while ignoring otolith dynamics. More advanced models could discriminate regular and irregular afferents taking into account non-linearities and the stochastic nature of vestibular motion perception (98).

The biomechanical neck model contains detailed structures leading to a high computational demand, taking days to fit experimental datasets. To enable individual modeling, we explore computationally efficient (simplified) biomechanical neck and full body models running faster than real time and explore optimal control strategies to explain and predict modulation of postural stabilization.

## Data availability statement

The raw data supporting the conclusions of this article will be made available by the authors, without undue reservation.

## Ethics statement

Ethical approval was not required for the study involving humans in accordance with the local legislation and institutional requirements. Written informed consent to participate in this study was not required from the participants or the participants’ legal guardians/next of kin in accordance with the national legislation and the institutional requirements.

## Author contributions

RH: Conceptualization, Data curation, Funding acquisition, Methodology, Validation, Writing – original draft. VK: Data curation, Formal analysis, Investigation, Software, Writing – review & editing. KW: Methodology, Writing – review & editing.

## Footnotes

^1^Similar models may address vestibular-ocular reflexes but this is beyond the scope of the current paper.
