# Supplementary material for: Neck stabilization through sensory integration of vestibular and visual motion cues
Source: Front Neurol. 2023 Nov 23;14:1266345. doi: 10.3389/fneur.2023.1266345 (PMC10704035; doi:10.3389/fneur.2023.1266345)
Supplement: Supplementary file 1 [file Data_Sheet_1.pdf]

APPENDIX (online) for the paper

Happee R, Kotian V and De Winkel KN (2023)

Neck stabilization through sensory integration of vestibular and visual motion cues.

Front. Neurol. 14:1266345. doi: 10.3389/fneur.2023.1266345

Neck, Vestibular & Muscle Model Equations and Parameters

As outlined in Fig. 1 the activation of muscle segments was regulated by vestibular/visual and proprioceptive afferent feedback, as well as neck muscle co-contraction. The neural signals from the vestibular/visual and muscle spindle pathways, together with co-contraction and postural activity were summed to define a neural excitation e for each muscle segment constrained between zero and one (from no to full activation). Muscle active state a responds to e with nonlinear dynamics (Eq. A1) with rapid activation (T_{ac}=10 ms time constant) and slower deactivation (T_{da} =40 ms time constant):

$$H_{act} = \begin{cases} \frac{1}{T_{ac}s+1} & e > a \; (activation) \\ \frac{1}{T_{da}s+1} & e \leq a \; (deactivation) \end{cases} \qquad \text{(A1)}$$

Muscle contraction dynamics were defined using a nonlinear Hill-type muscle model (1-3) with equations described in (4) and parameters given in Table A1. A nonlinear series elastic (SE) element was added in Matlab-Simulink as described in (5). The parameter SE_{xm} representing elongation at maximum isometric force was selected to be 10% which improved the model fit at higher frequencies compared to general values of 4-7% elongation for muscle tissue (3). This higher SE_{xm} can be motivated by the higher maximum isometric muscle stress (70 N/cm^2) in our model compared to 50 N/cm^2 in Winters and Stark (3). The maximum relative force during lengthening CE_{ml} was enlarged to 2.3 based on (6). The resulting functions describing muscle force as a function of length and velocity are shown in Fig. A1.

Linear damping coefficients for translation and rotation of the intervertebral discs were initially selected and validated for impact simulations (7). To improve the model fit in all conditions disc rotational damping was reduced from 0.5 to 0.3 Nm/rad while translational damping was reduced from 1000 to 800 Ns/m for shear and increased from 1000 to 1600 Ns/m for compression/elongation. The latter change was essential to prevent oscillations around 11 Hz and will benefit from targeted validation with a higher bandwidth and with vertical loading.

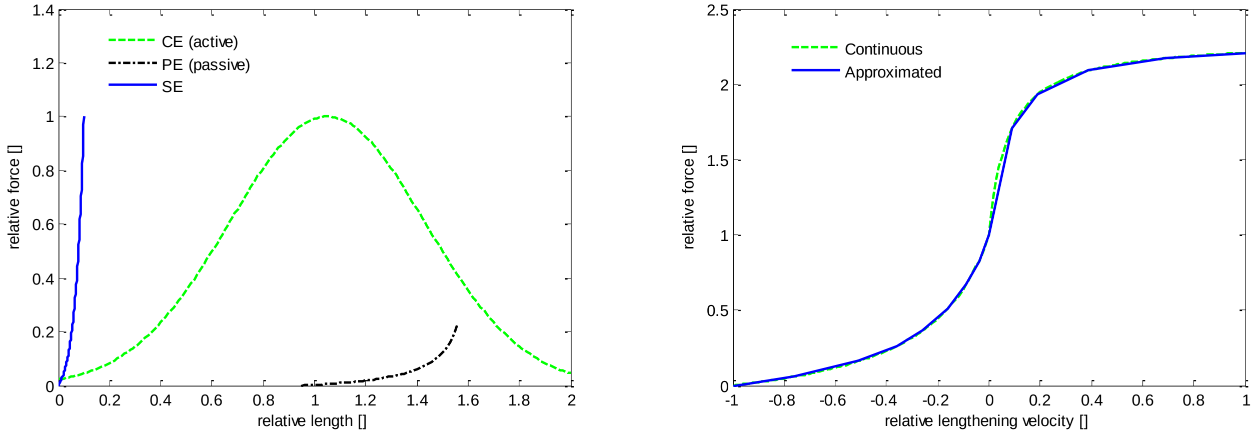

Fig. A1 Hill muscle model functions describing muscle relative force as a function of CE length (left) and velocity (right). The force velocity curve was approximated using a lookup table to facilitate inversion.

Table A1 Hill type muscle model parameters for active and passive behaviour, delays and postural feedback parameters.

Muscle parameters	Symbol	Unit	Value		References and remarks
			Original (8)	Current	
CE length at reference position	L_r	m			derived from the model geometry
CE optimum length	L_{opt}	m	1.05 L_r		de Bruijn, van der Helm (4), van der Horst (7), de Jager (9)
CE maximum shortening velocity	v_{max}	m	6 L_{opt}		Winters and Stark (3)
maximal isometric stress	σ_{max}	N/cm^2	70		Wood, Meek (10), Karlsson and Peterson (11), De Duca and Forrest (12)
shape force-velocity curve (shortening)	CE_{sh}	-	0.25		Winters and Stark (3), Bottinelli, Canepari (13)
shape force-velocity curve (lengthening)	CE_{shl}	-	0.075		Winters and Stark (3), Bottinelli, Canepari (13)
maximum relative force (lengthening)	CE_{ml}	-	2.3		Cole, van den Bogert (6)
shape active force-length curve	S_k	-	0.54		Winters and Stark (3)
SE elongation with maximum isometric force	SE_{xm}	-	0.1		Winters and Stark (3), see text
SE shape parameter	SE_{sh}	-	3		Winters and Stark (2)
passive force-length stiffness	k	N/cm^2	3.34		Deng and Goldsmith (14)
passive force-length asymptote	a	-	0.7		van der Horst (7), Deng and Goldsmith (14)
Delays and postural feedback parameters					
activation time constant	T_{ac}	s	0.010		Winters and Stark (2)
deactivation time constant	T_{da}	s	0.040		Winters and Stark (2)
cervicocollic (muscle) feedback delay	τ_{ccr}	s	0.018		Peterson, Goldberg (15)
vestibulocollic feedback delay for all vestibular and visual contributions	τ_{vcr}	s	0.013		Colebatch, Halmagyi (16), (17)
additional time constant for angular feedback (first order low pass) also taking into account visual delays	τ_{ang}	s	-	0.030-0.400	Model fitting (see text)
weighting of semicircular irregular fibres in head rotation rate feedback	kir	-	0.5	0.8	
weighting of otolith irregular fibres in head translation acceleration feedback	kit	-	0.5	na	Not used in the current paper, where otolith dynamics are ignored in line with published MSOM and SVC models
k_v during shortening	k_{vs}	-	0	k_v	see text

Vestibular feedback

Vestibulocollic afferent feedback is evoked by the semicircular canal and otolith organs located within the vestibular system. We adopted vestibular sensor dynamics based on experiments with macaque monkeys with naturalistic head motion ranging from low to high amplitude (up to 1500 deg/s and 8 G), where extracellular single-unit activity of semicircular canal and otolith afferents was recorded using glass microelectrodes (18). The semicircular canal organs primarily detect head angular velocity ($\dot{\theta}$). Linearized (low amplitude) canal afferent dynamics were adopted from (18) as:

$$H_{canal} = k\frac{s(s+1/T_1)}{(s+1/T_c)(s+1/T_2)} \qquad \text{(A2)}$$

For regular afferents, parameter values were: k = 2.83 (spikes/s)/(deg/s), T_1 = 0.0175 s, T_2 = 0.0027 s, and T_c = 5.7 s. For irregular afferents, parameter values were: k = 27.09 (spikes/s)/(deg/s), T_1 = 0.03 s, T_2 = 0.0006 s, and T_c = 5.7 s.

For the semicircular canal, we assumed a weighted contribution of regular and irregular afferents to head-neck stabilization. In the original paper (8) we assumed an equal weighting (kir=0.5) and in the current paper a higher weighting was applied to the irregular canal afferents (kir=0.8) which improved the model fit for the most challenging cases.

In the original paper (8) otolith dynamics were based on equation 2 of Schneider, Jamali (18) and static tilt experiments by Fernandez (19), as illustrated in figure A2. The current paper ignores otolith dynamics and assumes a simple gain in line with the assumptions of published MSOM and SVC models of sensory integration. This largely concurs with the rather flat frequency response of the regular otolith afferents in Figure A2.

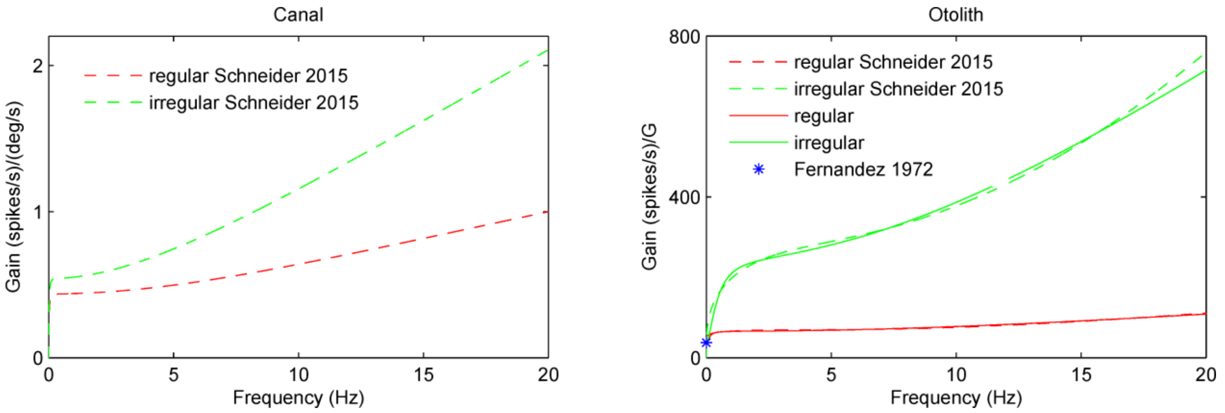

Fig. A2 Vestibular dynamics for the semicircular canals (left) and otolith organs (right).

As illustrated in Fig. 1, the three vestibular feedback components were multiplied with the feedback gain parameters $G_{ang.rate}$, G_{acc}, and G_{ang}. To convert the resulting control signals to an appropriate activation for individual muscles, muscle synergies were defined. Positive vestibular signals indicate forward deviation and activated extension muscle synergies $Na_{ext\text{-}r}$ and $Na_{ext\text{-}t}$, while negative vestibular signals indicated rearward deviation and activated flexion synergies $Na_{flex\text{-}r}$ and $Na_{flex\text{-}t}$ with subscripts r for rotation and t for translation control. These synergies were defined using isometric maximum voluntary contraction (MVC) simulations described by (4). The trapezius, levator scapulae, and rhomboid minor provided excessive extension and lateral moments. Hence, we disabled these scapular muscles in the isometric analyses to define muscle synergies, providing reduced head forces in extension (338 N) and lateral bending (194 N) close to isometric experiments (flexion force did not change).

To control head rotation using feedback of angular rate ($G_{ang.rate}$), the synergies $Na_{flex\text{-}r}$ and $Na_{ext\text{-}r}$ were derived generating head forward and rearward moments of 50% maximum voluntary contraction and scaled to 1 Nm.

The vestibulocollic feedback delay τ_{vcr} was set to 13 ms based on human studies (16, 17). This delay applies to all vestibular/visual feedback either applied as direct feedback of vestibular perception, or using sensory integration of vestibular and visual information. The additional time constant for angular feedback τ_{ang} (first order low pass) also taking into account visual delays was fitted per condition.

Feedback of the head angle in space (G_{ang}) was originally implemented driving the same synergies $Na_{flex\text{-}r}$ and $Na_{ext\text{-}r}$. This was effective for small perturbations, but not for larger perturbations (yaw & slalom). This was resolved by connecting feedback of the head angle in space (G_{ang}) to the muscle spindle feedback, dynamically adapting the desired muscle length L_0. Using this approach the muscle feedback loop mitigates the effects of nonlinear muscle dynamics.

To control head translation using acceleration feedback (G_{acc}), the synergies $Na_{flex\text{-}t}$ and $Na_{ext\text{-}t}$ were derived generating head forward and rearward forces of 50% maximum voluntary contraction and scaled to forces of 1 N at the head center of gravity (CoG). However this feedback loop is not addressed in the current paper, which focusses on head rotation control.

Muscle feedback

Muscle spindle afferent feedback was modelled using muscle CE length (*L*) and velocity ($\dot{L}$) feedback for each muscle element. Length and velocity were normalized to respectively the optimum length *(L_{opt})* and maximum shortening velocity (v_{max}) as defined in Table A1. Muscle CE length *L* was referenced to its desired value L_0 representing the desired posture which is dynamically adjusted based on the head angular feedback loop described above.
Based on a study on cats, the cervicocollic (muscle) feedback delay τ_{ccr} was set to 18 ms (15). Muscle spindles are particularly sensitive to positive (i.e. muscle lengthening) rather than negative velocities of stretch (20). This nonlinear velocity sensitivity was initially (8) modelled using half-wave rectification (21, 22). This was successful in most applications but in the slalom simulations, high CCR feedback gains were needed to fit the experimental data resulting in oscillations around 10 Hz. A reduction of the CCR feedback delay resolved these oscillations, where a substantial improvement was obtained lowering the delay from 18 to 10 ms and oscillations vanished with 1 ms. These oscillations also reduced very strongly when the half-wave rectification on spindle velocity was removed. Hence, the CCR delay was restored to the plausible value of 18 ms and the half-wave rectification was not further applied. Thereby the velocity sensitivity during shortening (k_{vs}) now equals the sensitivity during lengthening (k_v).
The gains controlling normalized muscle position *(k_p)* and velocity *(k_v)* feedback were initially (8) chosen identical for all muscle segments. This resulted in some imbalance since the neck extensors can generate higher moments than the neck flexors. Hence all gains k_p and k_v for neck flexors were scaled up with a factor 1.4.
Muscle spindle density is extremely high in the neck (23) such that small motions can accurately be sensed. Based on studies of cat neck muscles the Golgi contribution was, in contrast to the muscle spindles, found not to be substantial at low levels of tension (24). Because the current study investigated head motion close to the neutral position, and therefore likely induced low levels of muscle tension, Golgi feedback was not modelled.

Co-contraction and postural activity

The neck model comprises 24 rotational degrees of freedom complicating the definition of co-contraction (a review of co-contraction definitions can be found in (25)). We adopted a generic approach activating all neck muscles in a balanced manner enforcing a minimum activation. Muscle co-contraction was modified using the parameter G_{cc} and recruited the muscle synergy Na_{cc} which was derived using isometric analysis. A zero load task without gravity was simulated imposing zero joint moments while recruiting all muscles with at least 10%, which resulted in 14% activation averaged over muscles. The co-contraction synergy Na_{cc} was derived scaling this set of muscle activations down to an average of 1% and a minimum of 0.7%. Thereby a G_{cc} of 1% provides an average muscle activation of 1% and generates zero moments in all joints. An additional postural activity Na_{post} counteracting gravity for the selected initial posture was derived activating mainly extensor muscles.

Sensory integration models

See (26) for details and Table 1 for parameters.

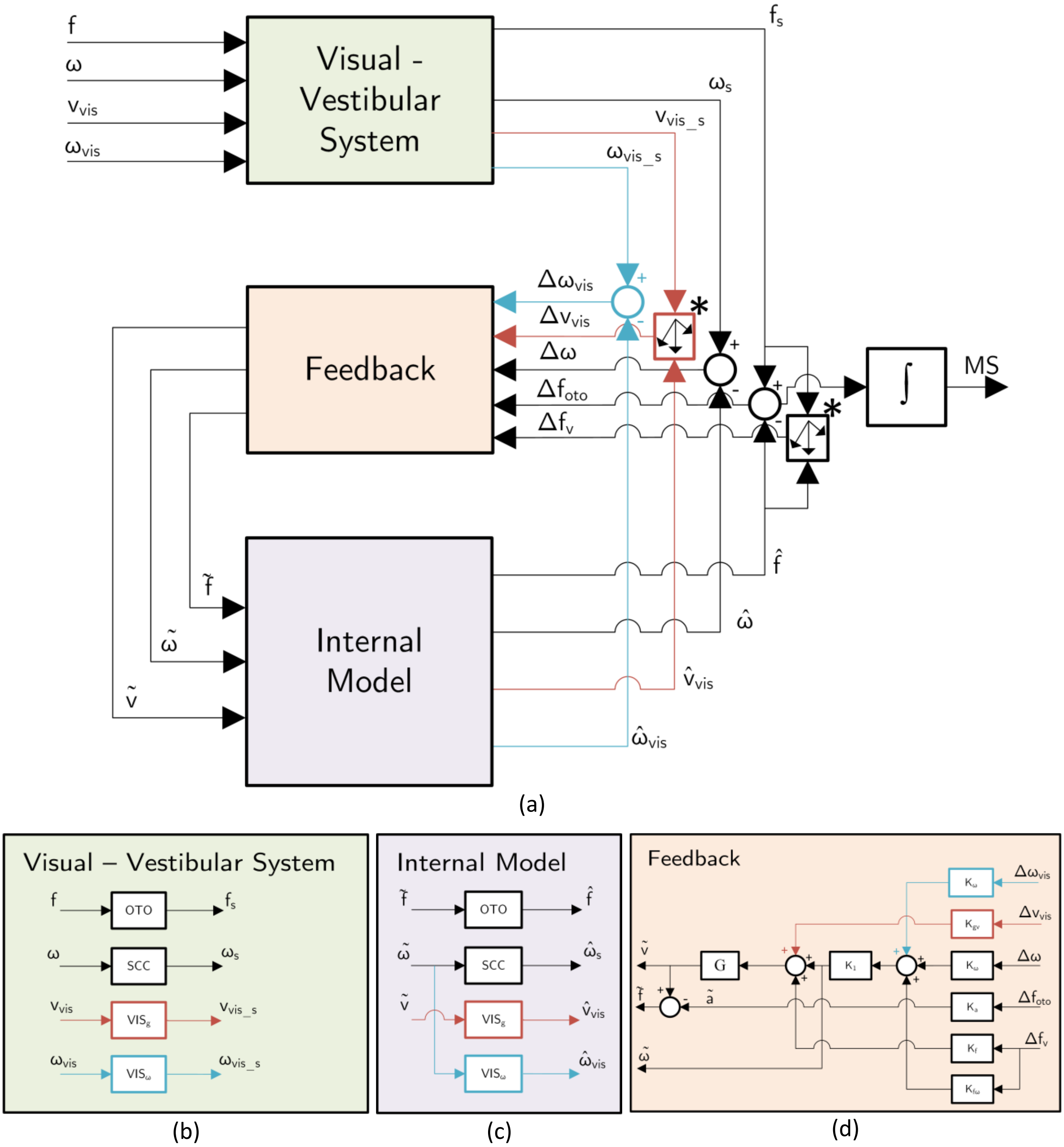

Fig. A3 Multisensory observer model (MSOM) redrawn after (27) .
(a) high-level diagram and (b)-(d) details of different subsystems. Vestibular loops (black); `VR' (visual rotational rate) loops (blue); `VV' (visual vertical (direction of gravity or orientation)) loops (red).
∗ *calculates the rotation required to align the actual and expected sensory estimates (see* (27) *for more details). The G block calculates the subjective vertical (direction of gravity)* (27)*. The otolith conflict* Δf_{oto} *is used for motion sickness (MS) prediction as motivated in* (26) through integration in time. Pitch and roll are estimated (equation 1) using the verticality estimate ṽ.

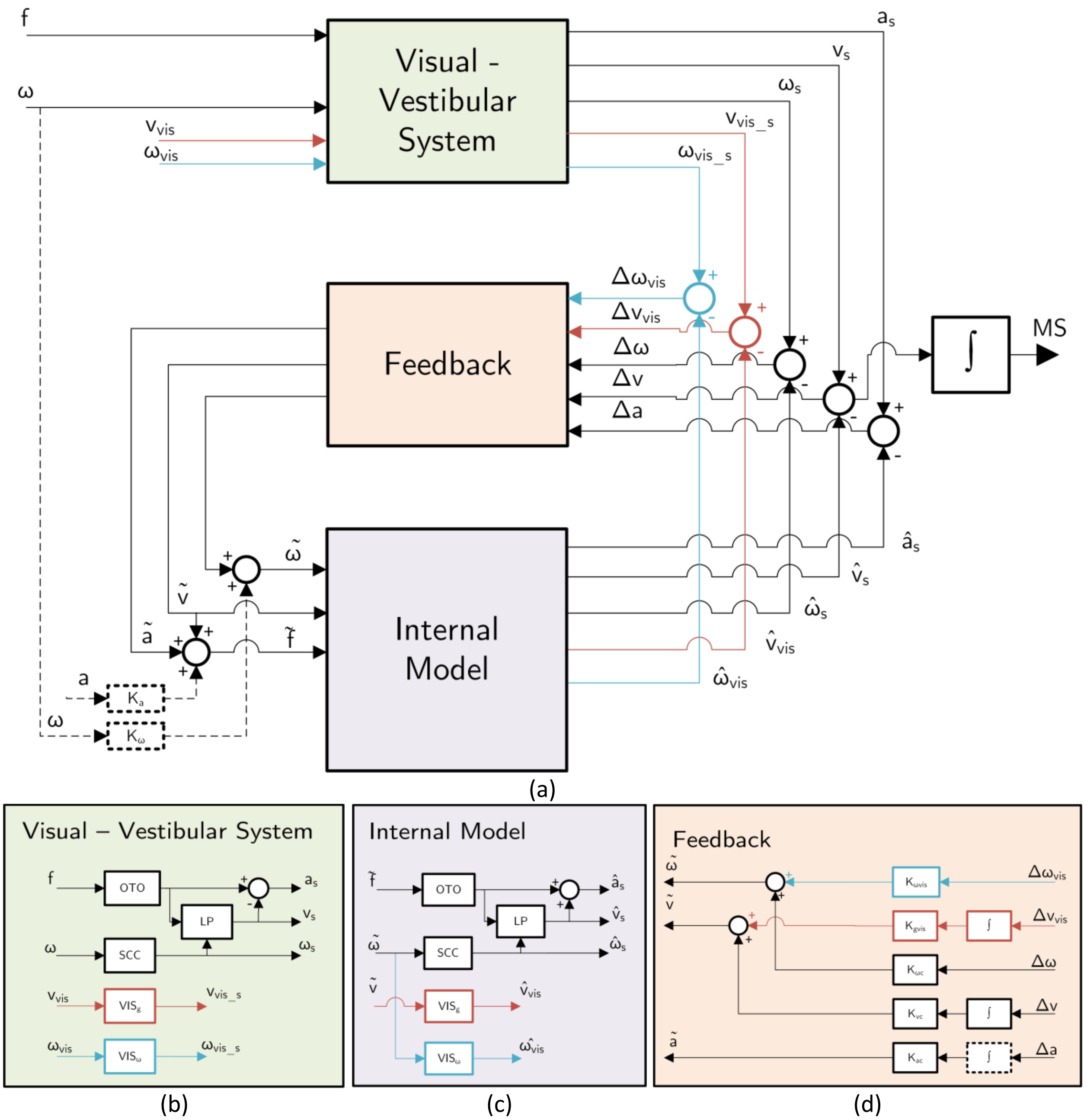

Fig. A4 Subjective vertical conflict (SVC) model, redrawn after (28-30).
(a) high-level diagram and (b)-(d) details of different subsystems. Vestibular loops (black) are as in (30); `VR' (visual rotational rate) loops (blue), as in (29); `VV' (visual vertical (direction of gravity or orientation)) loops (red), as in (28). As motivated in (26) we use the so-called SVC_I model which contains an integrator (dotted box in diagram d) to process Δa. The verticality conflict Δv is used for motion sickness (MS) prediction through integration in time. Pitch and roll are estimated (equation 1) using the verticality estimate $\tilde{v}_s$ in SVC_{int} and v_s in SVC_{vest}. The anticipatory gains K_a and K_ω are chosen to be zero in this study as we only study passive motion.

Error criterion for model correspondence with experimental data

To assess the model correspondence with the human response data, and to estimate (tune) the model parameters an error criterion *E* was defined. This criterion captures the model fit in the frequency domain using gain and phase, except for the slalom where head rotation signals (root mean square) in the time domain were used.
The frequency domain criterion sums the root mean square gain error EG over *nf* frequencies as the difference between the model gain and the experimental gain:

$$EG = \sqrt{\frac{1}{nf}\sum_{i=1}^{nf}\left(\frac{Gain_{mod}\,(i) - Gain_{exp}\,(i)}{Gain_{exp}\,(i) + 0.05\,Gain_{av}}\right)^2} \qquad \text{(A3)}$$

This error is defined relative to the experimental gain plus a small term being 0.05 times the average gain over all frequencies (*Gain*$_{av}$) which was added to reduce the weight on frequencies with very low gains. For some cases an additional weight was applied on the lowest frequencies as the data contained relatively few low frequencies.

For phase (in deg) a similar criterion EP was defined as:

$$EP = \sqrt{\frac{1}{nf}\sum_{i=1}^{nf}\left(\frac{Phase_{mod} - Phase_{exp}}{90}\right)^2} \qquad \text{(A4)}$$

where the error was scaled towards 90 deg. Finally the average error E was derived averaging over the relevant signals and averaging *EG* and *EP*. Thus the average error ranged between zero and around one, where one represents very poor gain fits, and phase errors around 90 deg.

References

1. Hill AV. The heat of shortening and the dynamic constants of muscle. Proceedings of the Royal Society Series B-Biological Sciences. 1938;126(843):136-95.
2. Winters JM, Stark L. Analysis of fundamental human movement patterns through the use of in-depth antagonistic muscle models. IEEE Trans Biomed Eng. 1985;32(10):826-39.
3. Winters JM, Stark L. Estimated mechanical properties of synergistic muscles involved in movements of a variety of human joints. J Biomech. 1988;21(12):1027-41.
4. de Bruijn E, van der Helm FCT, Happee R. Analysis of isometric cervical strength with a nonlinear musculoskeletal model with 48 degrees of freedom. Multibody System Dynamics. 2015;36(4):339-62.
5. Happee R. Time optimality in the control of human movements. Biol Cybern. 1992;66(4):357-66.
6. Cole GK, van den Bogert AJ, Herzog W, Gerritsen KG. Modelling of force production in skeletal muscle undergoing stretch. J Biomech. 1996;29(8):1091-104.
7. van der Horst MJ. Human head and neck response in frontal, lateral, and impact loading. [PhD Thesis]: Eindhoven University of Technology; 2002.
8. Happee R, de Bruijn E, Forbes PA, van der Helm FCT. Dynamic head-neck stabilization and modulation with perturbation bandwidth investigated using a multisegment neuromuscular model. J Biomech. 2017;58:203-11.
9. de Jager MKJ. Mathematical Head-Neck Models for Acceleration Impacts. [PhD Thesis]: Eindhoven University of Technology; 1996.
10. Wood JE, Meek SG, Jacobsen SC. Quantitation of human shoulder anatomy for prosthetic arm control--I. Surface modelling. J Biomech. 1989;22(3):273-92.
11. Karlsson D, Peterson B. Towards a model for force predictions in the human shoulder. J Biomech. 1992;25(2):189-99.
12. De Duca CJ, Forrest WJ. Force analysis of individual muscles acting simultaneously on the shoulder joint during isometric abduction. J Biomech. 1973;6(4):385-93.
13. Bottinelli R, Canepari M, Pellegrino MA, Reggiani C. Force-velocity properties of human skeletal muscle fibres: myosin heavy chain isoform and temperature dependence. The Journal of physiology. 1996;495 (Pt 2)(2):573-86.

14. Deng YC, Goldsmith W. Response of a human head/neck/upper-torso replica to dynamic loading--II. Analytical/numerical model. J Biomech. 1987;20(5):487-97.
15. Peterson BW, Goldberg J, Bilotto G, Fuller JH. Cervicocollic reflex: its dynamic properties and interaction with vestibular reflexes. J Neurophysiol. 1985;54(1):90-109.
16. Colebatch JG, Halmagyi GM, Skuse NF. Myogenic potentials generated by a click-evoked vestibulocollic reflex. Journal of neurology, neurosurgery, and psychiatry. 1994;57(2):190-7.
17. Forbes PA, Siegmund GP, Happee R, Schouten AC, Blouin JS. Vestibulocollic reflexes in the absence of head postural control. J Neurophysiol. 2014;112(7):1692-702.
18. Schneider AD, Jamali M, Carriot J, Chacron MJ, Cullen KE. The increased sensitivity of irregular peripheral canal and otolith vestibular afferents optimizes their encoding of natural stimuli. J Neurosci. 2015;35(14):5522-36.
19. Fernandez C, Goldberg JM, Abend WK. Response to static tilts of peripheral neurons innervating otolith organs of the squirrel monkey. J Neurophysiol. 1972;35(6):978-87.
20. Grill SE, Hallett M. Velocity sensitivity of human muscle spindle afferents and slowly adapting type II cutaneous mechanoreceptors. The Journal of physiology. 1995;489 (Pt 2)(2):593-602.
21. Stein RB, Kearney RE. Nonlinear Behavior of Muscle Reflexes at the Human Ankle Joint. Journal of Neurophysiology. 1995;73(1):65-72.
22. Kearney RE, Hunter IW. System identification of human triceps surae stretch reflex dynamics. Exp Brain Res. 1983;51(1):117-27.
23. Liu JX, Thornell LE, Pedrosa-Domellof F. Muscle spindles in the deep muscles of the human neck: a morphological and immunocytochemical study. J Histochem Cytochem. 2003;51(2):175-86.
24. Richmond FJ, Abrahams VC. Physiological properties of muscle spindles in dorsal neck muscles of the cat. J Neurophysiol. 1979;42(2):604-17.
25. Le P, Best TM, Khan SN, Mendel E, Marras WS. A review of methods to assess coactivation in the spine. J Electromyogr Kinesiol. 2017;32:51-60.
26. Kotian V, Irmak T, Pool DM, Happee R. The role of vision in sensory integration models for predicting motion perception and sickness. Experimental Brain Research. under revision.
27. Newman MC. A Multisensory Observer Model for Human Spatial Orientation Perception [PhD thesis]: Massachusetts Institute of Technology; 2009.
28. Liu H, Inoue S, Wada T. Motion Sickness Modeling with Visual Vertical Estimation and Its Application to Autonomous Personal Mobility Vehicles. 2022.
29. Wada T, Kawano J, Okafuji Y, Takamatsu A, Makita M, editors. A Computational Model of Motion Sickness Considering Visual and Vestibular Information. 2020 IEEE International Conference on Systems, Man, and Cybernetics (SMC); 2020: IEEE.
30. Kamiji N, Kurata Y, Wada T, Doi Si, editors. Modeling and validation of carsickness mechanism. SICE Annual Conference; 2007: IEEE.
